# Comparison of deep LSTM and machine learning models for predicting compressive strength of fly ash/slag-based geopolymer concrete

**DOI:** 10.1038/s41598-025-14365-6

**Published:** 2025-09-25

**Authors:** Ceren Kina, Harun Tanyildizi, Mohd Mustafa Al Bakri Abdullah, Rafiza Abdul Razak, Thanongsak Imjai

**Affiliations:** 1 Faculty of Engineering and Natural Sciences, Department of Civil Engineering, Malatya Turgut Ozal University, Malatya, Türkiye; 2 Technology Faculty, Department of Civil Engineering, Firat University, Elazig, Türkiye; 3 Center of Excellence Geopolymer & Green Technology (CEGeoGTech), Universiti Malaysia Perlis (UniMAP), Perlis, 01000 Malaysia; 4 Faculty of Chemical Engineering & Technology, Universiti Malaysia Perlis (UniMAP), Perlis, 01000 Malaysia; 5 Faculty of Civil Engineering & Technology, Universiti Malaysia Perlis (UniMAP), Perlis, 01000 Malaysia; 6 School of Engineering and Technology, Walailak University, 222 Thaiburi, Thasala, 80160 Nakhon Si Thammarat Thailand

**Keywords:** Geopolymer concrete, Ground granulated blast furnace slag, Fly ash, Machine learning, Compressive strength, Engineering, Materials science

## Abstract

In the production of geopolymer concrete (GPC), using ground granulated blast furnace slag (GGBFS) and fly ash (FA) can reduce the carbon dioxide footprint and decrease the amount of waste materials released into the environment. Finding the compressive strength (fc) of GPC through experiments is time-consuming and costly; thus, applying artificial intelligence models can expedite this process. This study aims to compare the performance of deep Long Short-Term Memory (LSTM) and the machine learning (ML)-based algorithms in predicting the f_c_ of FA/GGBFS-based GPC. Artificial neural networks (ANN), Bootstrap aggregating (Bagging), Least-Squares Boosting (LSBoost) and K-Nearest-Neighbours (kNN) were used for ML-based algorithms. For this goal, data were collected from the previous studies in the literature. The selected input characteristic variables included the chemical composition and quantities of FA and GGBFS, fine and coarse aggregates, sodium hydroxide molarity, alkaline activators, superplasticizer dosage, and curing temperature. Based on sensitivity analysis, the most influential parameter in the f_c_ of FA/GGBFS-based GPC was the fine aggregate content. Performance metrics, error percentage distribution, and Taylor diagrams indicate that the highest accuracy was achieved by LSTM, which had an R-squared value of 0.98. This was followed by ANN, LSBoost, Bagging, and kNN. Notably, LSBoost and ANN also demonstrated strong performance, with R-squared values of 0.94 and 0.95, respectively. Also, Bagging showed acceptable ability for f_c_ estimation of FA/GGBFS-based GPC due to having an R-squared value of 0.88, but kNN had very poor performance.

**Supplementary Information:**

The online version contains supplementary material available at 10.1038/s41598-025-14365-6.

## Introduction

### Background

Recently, there has been a growing awareness regarding the environmental impact of concrete as a basic building material. The need for raw materials is increasing due to the rising global population. Estimates suggest that the global concrete and cement market will double by 2050, leading to higher carbon emissions and further damage to biodiversity^1^. Researchers are focusing on the development of alternative binders to replace Portland Cement (PC) as it requires significant energy during production and has a substantial carbon footprint. The production of PC, the main binding component in concrete, demands approximately 1.7 tons of raw materials, resulting in the emission of about 0.8 tons of CO_2_^2^. These circumstances require rapid action to mitigate the adverse impacts of PC production against climate change. A promising scientific alternative is the recycling of industrial and agricultural wastes, which has natural compositions that can be used as building materials, promoting material sustainability^3^. The utilization of these wastes as a partial or complete substitute for PC as a binder can positively impact both the economy and environment by reducing carbon footprints^4–6^.

### Fly ash/slag-based geopolymer concrete

In recent years, there has been great demand for the production of high-performance, environmentally friendly, and low-cost building materials that can withstand adverse environmental conditions. Geopolymer concrete (GPC) is a type of environmentally friendly concrete that replaces the total dosage of PC with alternative components, such as recycled agro-industrial materials^7^. Its production involves activating aluminosilicate-based source materials with alkali hydroxide/alkali silicate. In addition to these aluminosilicate source binder materials, GPC also includes fine/coarse aggregate, alkaline solution, and water^8^. Moreover, most research demonstrates the utilization of recycled industrial and agricultural materials as promising geopolymer precursors, including GGBFS^9^ FA^10,11^ red mud (RM)^12^rice husk ash (RHA)^12^silica fume (SF)^13^ and metakaolin (MK)^14^. In the manufacturing process of GPC, alkaline activators such as sodium silicate (Na_2_SiO_3_), sodium hydroxide (NaOH), potassium silicate (K_2_SiO_3_), and potassium hydroxide (KOH) are used to activate the aluminosilicate materials^15^. The enhanced durability and mechanical strength of GPC make it an appropriate choice for use in environmentally sensitive areas^16^. Therefore, according to the literature, GPC exhibits improved mechanical and durability properties with regards to PC-based concrete because of its unique chemical texture^15,16^.

There are researches in the literature demonstrating the application of waste materials and recycled materials as promising geopolymer precursors, including SF, RHA, GGBFS, FA, MK, and RM^10–12,17^. The use of these residual recycled materials in GPC helps prevent them from ending up in landfills, thereby reducing their negative impact on the environment^18^. These materials are classified as binders and used as supplementary cementitious materials. The two most commonly used materials that show promise in the reduction of environmental impact are FA and GGBFS. FA-based GPC can reduce CO_2_ emission by up to 25–45% by employing waste coal combustion products^19^. It is also a cost-effective material that exhibits similar mechanical properties and comparable texture and appearance relative to traditional concrete^20^. Besides, it shows improved durability, including resistance to chloride and sulfate penetration and high-temperature resistance^21^. In contrast to traditional concrete, GGBFS-based GPC also yields cost benefits, reduces adverse environmental impact^22,23^ and shows high resistance to chemical attacks^24^ along with increased stiffness^25^. The researchers have found that by integrating FA and GGBFS with alkaline solvents, such as sodium silicate and sodium hydroxide, it is possible to produce environmentally and economically viable GPC with comparable strength.

Researchers should explore mixed design options and suitable component selections to achieve the desired mechanical properties of GPC under laboratory conditions. However, the mix design of GPC is quite complex due to the numerous variables involved^26^. The NaOH concentration and its ratio with Na_2_SiO_3_^27^, liquid-to-binder ratio^28^curing temperature^29^the Al and Si concentration in constituents^14^ influence the hardened properties of GPC. Additionally, variations in the chemical compositions and component proportions also affect the mix design of GPC. Achieving the required strength and durability response of GPC mix requires more trial mixes than cement-based concrete. Although the current practice of proportioning GPC components is inspired by the standard cement-based concrete mix design code, achieving optimal proportions often requires repetitive paths. Therefore, it is necessary to create a method that minimizes effort, energy, and time in the mix design process for GPC. This study utilized various parameter, including chemical compositions and amounts of FA and GGBFS, fine and coarse aggregate, NaOH, Na_2_SiO_3_, and SP, NaOH molarity and curing temperature. The focus of this research was to forecast the compressive strength of geopolymer composite using artificial intelligence methods, including LSTM, ANN, Bagging, LSBoost, and kNN.

### Data-driven methods

In recent years, advancements in modern computational resources have enabled the use of artificial intelligence, including deep learning (DL) and supervised machine learning (ML) algorithms and ensemble approaches across various fields^11–20^. These technologies are increasingly being applied to predict the mechanical properties of various material types^30^. ML approaches such as classification, regression, and clustering are utilized to forecast the strengths with high accuracy^31^. One of the most popular applications is predicting the mechanical properties of different types of concrete, as obtaining these results through traditional laboratory methods can be time-consuming, expensive, and energy-intensive. By the utilization of ML techniques for the strength prediction of materials, several advantages arise, such as lower costs, simplicity, and speed, while maintaining higher accuracy compared to traditional experimental studies^32,33^. In addition, ensemble learning methods are frequently employed to achieve prediction accuracy by combining multiple base learners^34^. Deep learning methods address the limitations of hand-crafted features in complex ML programs. Its several network layers can increase the nonlinear mapping capability with regard to ML-based algorithms. DL algorithms can assist in the feature extraction process, allowing them to identify the characteristics necessary for achieving the desired output. Therefore, DL models can establish a direct mapping from the primary inputs to the target output with appropriate training without removing any features^35^. Thus, DL algorithms can break down complex tasks into simple problems^36,37^.

ML techniques enable machines to examine a sufficient number of data samples to obtain the information they need to perform a particular task^37^. Before using a method, it is necessary to perform feature extraction to identify the most informative data characteristics. The sample data used in the next stage of the procedure is based on a specific training strategy^36^. An analysis of several studies reviewed by Kumar et al.^3^ demonstrated that the selection of the forecasting model is influenced by the relationship between the mechanical strength and the components of concrete. The most widely used ML algorithms for the concrete strength prediction are support vector machine (SVM), ensemble of trees, gene expression programming (GEP), artificial neural networks (ANN), extended gradient boosting (XGB), etc^38–40^. Bagging and boosting algorithms are the ensemble learning approaches^41^. Bagging series algorithms can perform parallel operations on base learners independently and then combine them into an ensemble strategy, while boosting series algorithm can perform serial operations on base learners^42^. There are four major deep learning models: deep belief network (DBN), auto-encoder (AE), convolutional neural network (CNN), and recurrent neural network (RNN). The weakness of gradient disappearance for RNN can be solved by the three-gate structure of the Long Short-Term Memory (LSTM)^43^.

Numerous studies were carried out by the researchers to predict the f_c_ of various concrete types. Kumar et al.^1^ predicted the f_c_ of self-compacting concrete containing PC, FA and SF as binders using gradient boosting (GB), GB-particle swarm optimization, GB-Bayesian optimization, GB-differential evolution. In the other study conducted by Kumar et al.^2^, the f_c_ of ultra high performance concrete was predicted via recurrent neural networks, LSTM, and bi-LSTM. Sapkota et al.^6^ developed ensemble machine learning models, including random forest–krill herd, extended gradient boosting (XGB)-krill herd, XGB-leopard seal algorithm, and random forest-leopard seal algorithm to predict the f_c_ recycled plastic-based concrete. Shrestha and Sapkota^7^ predicted the f_c_ of ultrahighperformance concrete using random forest-pelican optimization algorithm, XGB-pelican optimization algorithm, random forest-walrus optimization algorithm, and XGB-walrus optimization algorithm. Sapkota et al.^8^ optimized the f_c_ of high-strength concrete using XGB model with hyperparameter optimization via cuckoo search, water strider, leopard seal, harris hawk, invasive weed, and forest optimization algorithms. Sapkota et al.^10^ also developed XGB with five optimization algorithms, namely random search, grid search, bayesian optimization, grey wolf optimization, and particle swarm optimization to predict the splitting tensile strength of recycled aggregate concrete.

Table 1 summarizes the literature review on the algorithms developed for the prediction of f_c_ of GPC blended with FA and/or GGBFS. They utilized different databases and several ML techniques.

**Table 1:** A review for the ensemble, DL/ML-based algorithm applications to predict f_c_ of GPC having FA/GGBFS.

Reference	Input parameters	Dataset (samples)	DL Algorithms	ML Algorithms
Nguyen et al.^44^	FA, water glass solution, NaOH solution, coarse and fine aggregate, water, NaOH solution concentration, curing time, curing temperature	335	DNN, DRN	–
Oyebisi et al.^45^	GGBFS, corncob ash, fine and coarse aggregate, water, NaOH pellets, sodium silicate gel, curing day, NaOH solution concentration, concrete grade	288	DNN	–
Shahmansouri et al.^13^	SF, natural zeolite, GGBFS, age, NaOH concentration	117	ANN	–
Khan et al.^46^	Alkali activator/FA, Na_2_SiO_3_/NaOH, curing temperature, curing time, age, NaOH solution molarity, percent SiO_2_ solids/water, plasticizer percent, water, percent volume of total aggregate, fine aggregate/total aggregate	298	–	GEP, RF
Shahmansouri et al.^47^	GGBFS, SF, natural zeolite, NaOH solution concentration, age	351	–	GEP
Kumar et al.^48^	FA, GGBFS, fine and coarse aggregate, alkali activator	60	CNN	RF, SVM,
Pazouki^10^	FA, fine and coarse aggregate, curing period, temperature, age, water, superplasticizer, molarity, Na_2_SiO_3_ and NaOH content	360	ANN	RBFNN, ACO, GMDH,
Kina et al.^9^	GGBFS, SF, natural zeolite, NaOH solution concentration, age	117	–	DT, Bagging, LSBoost
Ahmed et al.^49^	FA, GGBFS, alkaline solution/binder, fine and coarse aggregate, Na2SiO3 and NaOH content, SiO_2_/Al_2_O_3_ of FA, SiO_2_/CaO of GGBFS, Na_2_SiO_3_/NaOH, molarity	220	ANN	M5P
Nazar et al.^50^	FA, activator/FA, fine and coarse aggregate, mixing procedure, activator content, water, curing regime, molarity	245	ANN	ANFIS, GEP
Dao et al.^51^	FA, Na_2_SiO_3_ and NaOH content, water	210	ANN	ANFIS,
Khan et al.^52^	FA, fine and coarse aggregate, temperature and curing duration, molarity, alkaline activator/FA, water, Na_2_SiO_3_ and NaOH content	149	BPNN	RF, kNN
Ling et al.^53^	Liquid/FA, alkaline solution concentration, temperature, age, mole ratio,	273	ANN	–
Ahmad et al.^54^	FA, Na_2_SiO_3_ and NaOH content, NaOH molarity, curing age, fine and coarse aggregate, SiO_2_, Na_2_O	154	ANN	Boosting, AdaBoost
Khalaf et al.^55^	FA, coarse aggregate/total aggregate, Na_2_O, SiO_2_/Na_2_O, chemical composition index, geopolymer solids/total aggregate, water/geopolymer solid, superplasticiser, heat curing time and temperature	189	FLNN	–
Gupta et al.^56^	FA, superplasticiser, NaOH molarity, rest period, fine and coarse aggregate, NaOH/Na_2_SiO_3_, water, alkaline activator/binder	289	ANN	–
Tran^57^	FA, GGBFS, superplasticiser, water, fine and coarse aggregate, Na_2_SiO_3_ and NaOH content, curing period, rest period, curing temperature, NaOH/ Na_2_SiO_3_, alkaline activator/binder, molarity	158	–	SVM, RF, GB, AdaBoost, DT, kNN, GPR, CatB
Ahmed et al.^58^	GGBFS/binder, water, temperature, water/binder, fine and coarse aggregate, superplasticiser	268	–	SVM, GWO
Chu et al.^59^	FA, NaOH molarity, water, superplasticiser, percentage of total aggregate by volume, curing regime and time, age	311	–	GEP, MEP
Ahmad et al.^60^	FA, fine and coarse aggregate, Na2SiO3 and NaOH content, NaOH molarity, SiO2, Na2O	154	–	DT, Bagging, Adaboost
Zou et al.^61^	FA, GGBFS, Na_2_SiO_3_ and NaOH content, fine and coarse aggregate, NaOH molarity, water/solid	371	–	DT, GEP, Bagging, RF
Amin^62^	FA, GGBFS, fine and coarse aggregate, Na_2_SiO_3_ and NaOH content, superplasticiser, temperature	156	–	DT, SVR, DT-Adaboost, SVR-Adaboost, DT-Bagging, SVR-Bagging, RF
Sapkota et al.^8^	PC, FA, fine and coarse aggregate, water, superplasticiser, age, superplasticiser, alkali activators	689	–	AO-XGB, WS-XGB

*DNN* Deep Neural Network, *DRN* Deep Residual Network, *BPNN* Back-propagation neural network, *RF* Random Forest, *kNN* K-Nearest-Neighbours, *RBFNN* radial basis function neural network, *ACO* ant colony optimization, *GMDH* group method of data handling, *DT* decision tree, *LSBoost* Least-squares boosting, *M5P* M5P-Tree, *ANFIS* adaptive neuro-fuzzy inference system, *FLNN* feedforward layered neural network, *BPNN* backpropagation neural network, *GB* gradient boosting, *GB-BO* gradient boosting Bayesian optimization, *GB-PSO* gradient boosting particle swarm optimization, *GB-DE* gradient boosting differential evolution, *CatB* CatBoost, *GWO* Grey Wolf Optimization, *MEP* multi expression programming, *GPR* Gaussian process regression, *WS* Water Strider, *AO* Aquila optimizer.

## Research significance

In this study, an attempt has been performed for the prediction of the f_c_ of GPC blended with FA and GGBFS using prediction algorithms, namely Long Short-Term Memory (LSTM), Bootstrap aggregating (Bagging), Least-Squares Boosting (LSBoost), Artificial Neural Network (ANN) and K-Nearest-Neighbours (kNN). The significance and novelty of this study can be listed as follows:

- The literature reviews the effects of mineral admixtures—such as fly ash (FA), silica fume (SF), metakaolin, and ground granulated blast-furnace slag (GGBFS)—on the f_c_ of geopolymer concrete (GPC) through sensitivity analysis. A distinctive feature of this study is its focus on how the chemical compositions of these admixtures influence the development of eco-efficient GPC. In particular, the analysis considers the contents of SiO_2_, Al_2_O_3_, and CaO in FA and GGBFS for the first time.
- Considering Table 1, previous studies in the literature that applied ensemble, DL/ML-based models to predict the f_c_ of FA/GGBFS-based GPC have primarily focused on the ANN technique. Other algorithms devised in the current study, such as kNN, Bagging, and LSBoost have received less attention. Notably, deep LSTM networks have not been previously used to predict the f_c_ of GPC having FA/GGBFS as the binder. This research addresses the gap in the literature by comparing the commonly utilized ANN technique with these lesser-used algorithms (kNN, Bagging, LSBoost) and LSTM for the first time. Unlike traditional machine learning algorithms, which struggle with sequential data, LSTM approaches excel at learning and predicting future data sequences based on prior observations. Thus, this comparison is anticipated to provide valuable insights.
- In the current study, due to the wide use of FA and GGBFS in produced GPC, the samples having both of these two types of binders were taken into account while selecting the database. This approach was chosen to create a more comprehensive prediction model.
- The predictive model with high accuracy could assist researchers in evaluating the f_c_ of FA/GGBFS-based GPC more economically, quickly, and simplly than traditional experimental methods, which are often costly and time-consuming Developing five prediction algorithms could be beneficial for creating a highly accurate prediction model.

**Fig. 1:**
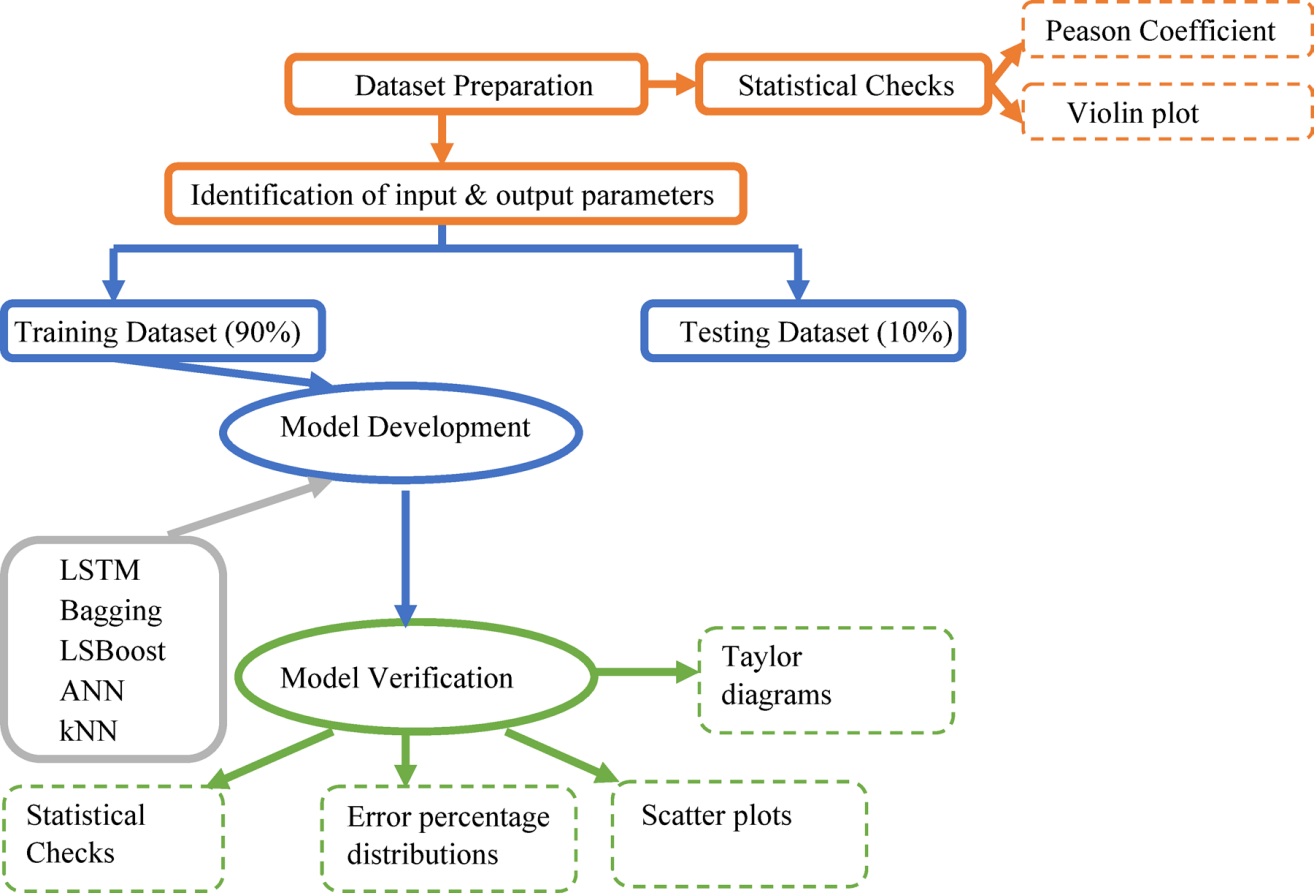
Flowchart of the proposed models.

The current study analyzed the f_c_ of FA/GGBFS-based GPC samples gathered from the previous studies^63–73^ via various ML-based algorithms. The motivation is to optimize the f_c_ prediction by comparing the results of different models using error percentage distribution, standard performance metrics, and Taylor diagramsto identify the most accurate model. The flowchart of the study was given in Fig. 1.

## Data preprocessing

**Fig. 2:**
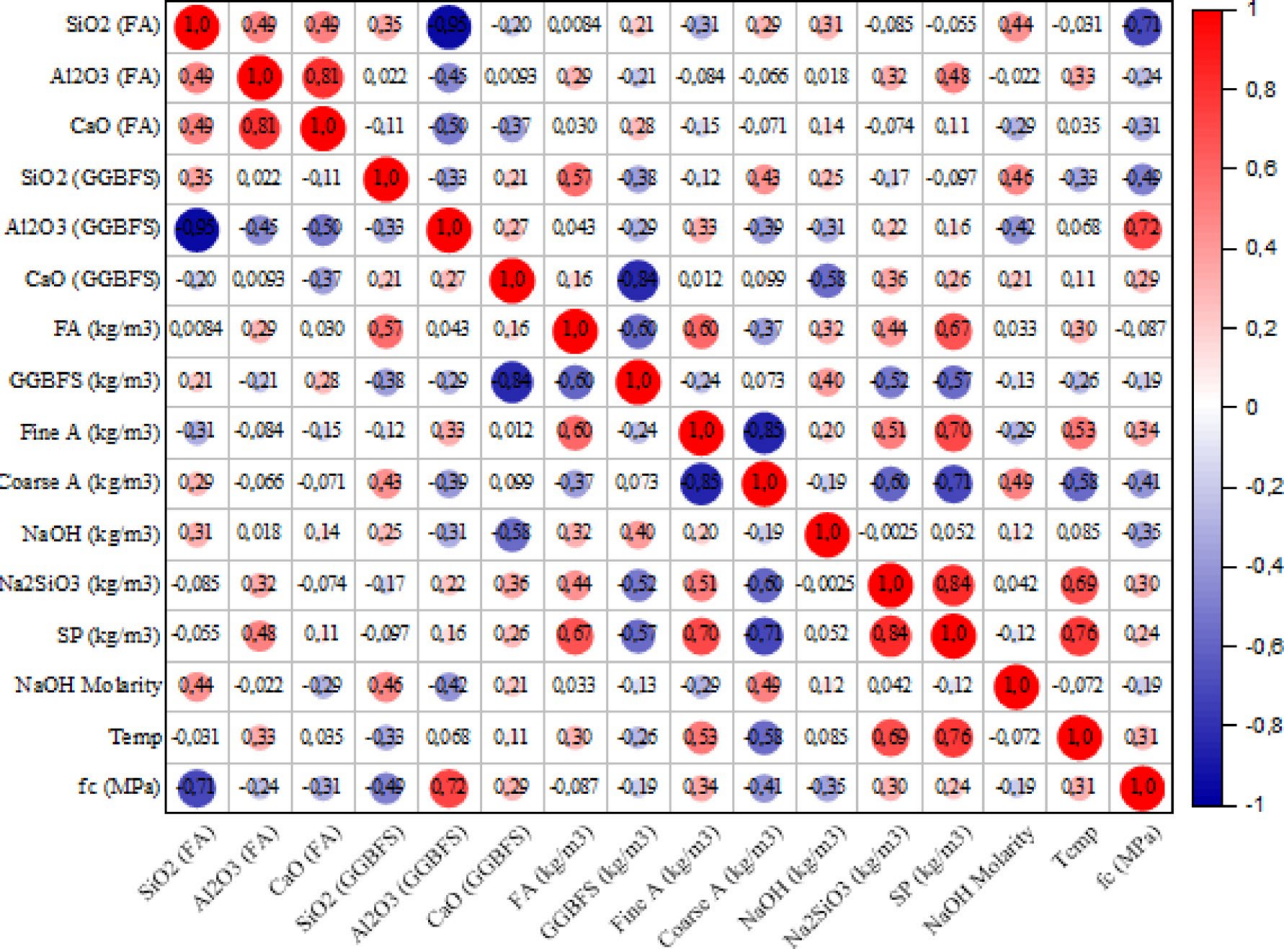
Pearson coefficient.

The dataset of GPC blended with FA/GGBFS was gathered from the open literature^63–73^ with 156 samples to develop ML-based algorithms for the f_c_ prediction. The details of the gathered dataset can be found in the Supplementary Material file. Although there are many studies using comprehensive dataset points to forecast the f_c_ of GPC, some researchers^9,48,52,54,57,60,62^ have used lower points ranging from 60 to 158 as given in Table 1, so in the current study, the dataset can be considered as sufficient. The studies having similar mixture contents were selected, and these experimental data were randomly selected to avoid bias. The input characteristic variables were selected as the amount and chemical content (SiO_2_, Al_2_O_3_, CaO) of FA and GGBFS, fine and coarse aggregate contents, sodium hydroxide (NaOH) molarity, alkaline activators, superplasticizer dosage (SP), and curing temperature. The output characteristic variable is the f_c_ of GPC. In other available studies on f_c_ prediction via different algorithms (see Table 1), the same input variables were utilized. The difference was that in some of them the researchers used the ratio of the mixture components such as Na_2_SiO_3_/NaOH^46,49,56,57^, fine aggregate/total aggregate^46^coarse aggregate/total aggregate^55^alkaline activator/binder^46,49,50,52,56,57^water/binder^58^. The statistical information of each variable was given in Table 2. In order to explore the relevance of each characteristic variable, the correlations between the variables were calculated. Pearson correlation coefficient (PCC) approach was applied to reveal the relationship between the characteristic variables. This approach evaluates the degree of linear relationships between features and the relative independence. It can be determined by the following equation:

1 $$\:{P}_{x,y}=\:\frac{COV(x,y)}{{\sigma\:}_{x}-{\sigma\:}_{y}}$$

where, $\:{P}_{x,y}$ is the Pearson correlation coefficient, $\:COV(x,y)$ is the coefficient of variation of x and y, $\:{\sigma\:}_{x}$, and $\:{\sigma\:}_{y}$ are the standard deviation. The closer the coefficient is to 1, the stronger the positive correlation between the variables. On the other hand, the closer the coefficient is to -1, the stronger the inverse correlation. Figure 2 shows the PCC plot as a matrix for all features. The correlation coefficients between the output (f_c_) and input variables are in the range of -0.71 and 0.72, which shows the complexity of the dataset. There is a nonlinear relationship between all the parameters, so these features cannot be discarded. The prediction models can be useful for processing these datasets. The analysis of the correlation indicates that the Al_2_O_3_ content in GGBFS showed a strong positive correlation of 72%, while the SiO_2_ content in FA revealed a negative correlation of -71%. These findings underscore the importance of considering how different variables affect the f_c_ of geopolymer concrete.

**Table 2:** Statistical descriptive of variables.

Variables	Type	Unit	Min^1^	Max^2^	Avg^3^	Std^4^	COV^5^	Kurtosis	Skewness
SiO_2_ (FA)	Input	%	0.0	400.0	252.5	86.3	0.3	2.5	− 1.4
Al_2_O_3_ (FA)	Input	%	0.0	65.6	52.8	13.6	0.3	9.3	− 3.0
CaO (FA)	Input	%	0.0	34.1	25.6	6.8	0.3	8.2	− 2.6
SiO_2_ (GGBFS)	Input	%	0.0	13.7	4.4	3.8	0.9	2.1	1.8
Al_2_O_3_ (GGBFS)	Input	%	0.0	409.0	151.4	86.7	0.6	2.2	1.3
CaO (GGBFS)	Input	%	21.0	37.7	33.1	3.6	0.1	5.5	− 2.3
FA	Input	kg/m^3^	0	400	252.5	86.3	0.3	2.5	− 1.4
GGBS	Input	kg/m^3^	0	409	151.4	86.7	0.6	2.2	1.3
Fine A.	Input	kg/m^3^	547	810.6	729.8	68.0	0.1	0.0	− 0.8
Coarse A.	Input	kg/m^3^	966	1293	1096.0	117.9	0.1	− 1.5	0.3
NaOH	Input	kg/m^3^	9	143.3	60.5	26.8	0.4	3.0	1.2
Na_2_SiO_3_	Input	kg/m^3^	54	192.9	123.0	35.7	0.3	− 0.9	0.1
SP	Input	kg/m^3^	0	180	77.6	81.0	1.0	− 1.9	0.2
NaOH	Input	M	0	16	8.6	3.9	0.5	0.2	− 0.5
Temp	Input	°C	0	60	28.1	20.6	0.7	− 0.9	0.3
f_c_	Output	MPa	10.5	89.6	42.7	15.3	0.4	0.6	0.6

^1^Minimum, ^2^Maximum, ^3^Average, ^4^Standard deviation, ^5^Coefficient of Variation.

**Fig. 3:**
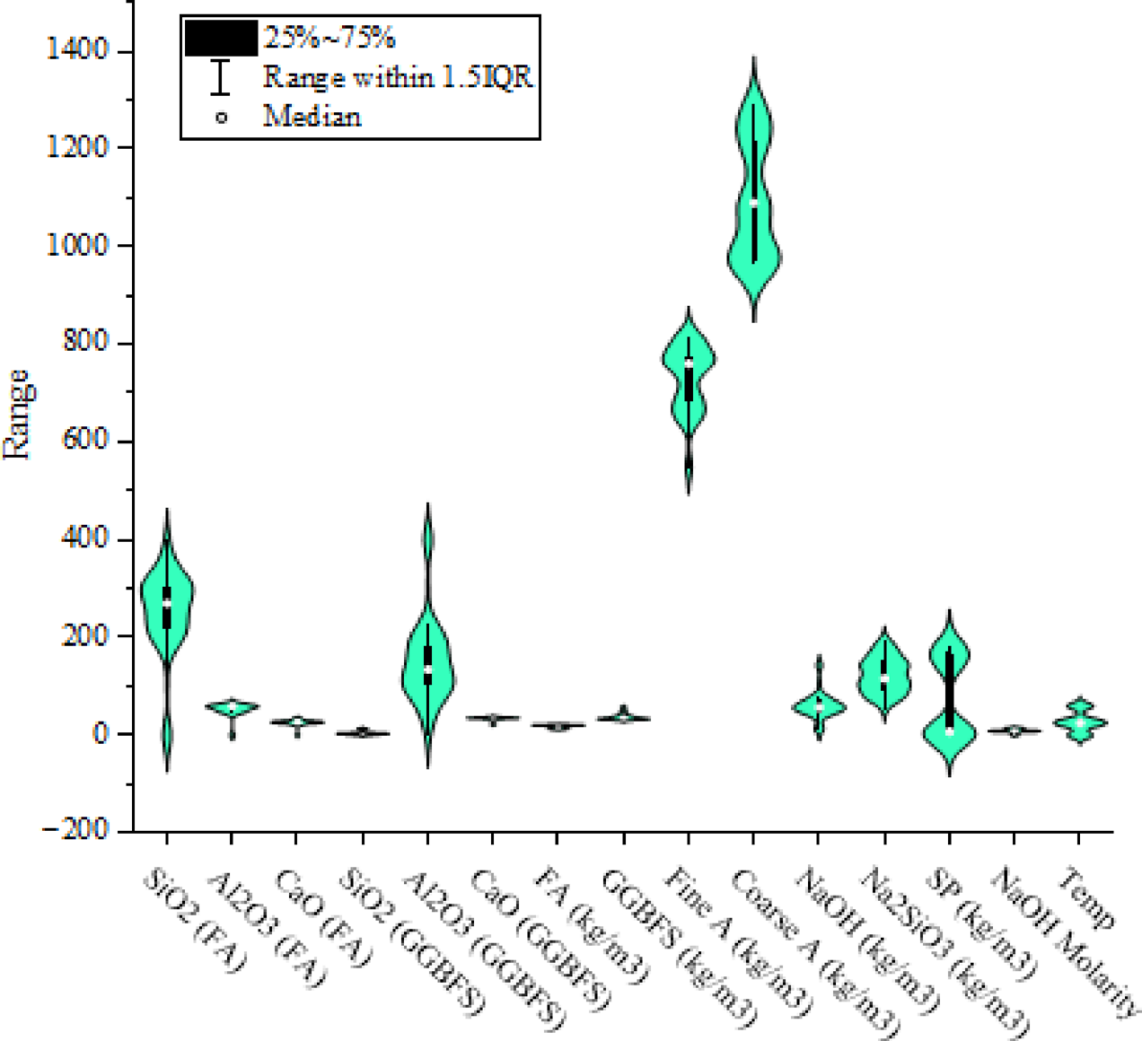
Violin plot of dataset.

Figure 3 displays the violin plot with box plots of the input parameters. This plot is utilized to visualize the distribution of a continuous variable. It highlights the minimum, maximum, median, first quartile, and third quartile of the dataset, which results in a more comprehensive representation of the data distribution. Observing Fig. 3, the distribution of SiO_2_ content in FA, Al2O3 content in GGBFS, fine and coarse aggregate contents, alkali activators, and SP were significant, while the other input parameters varied minimally.

**Fig. 4:**
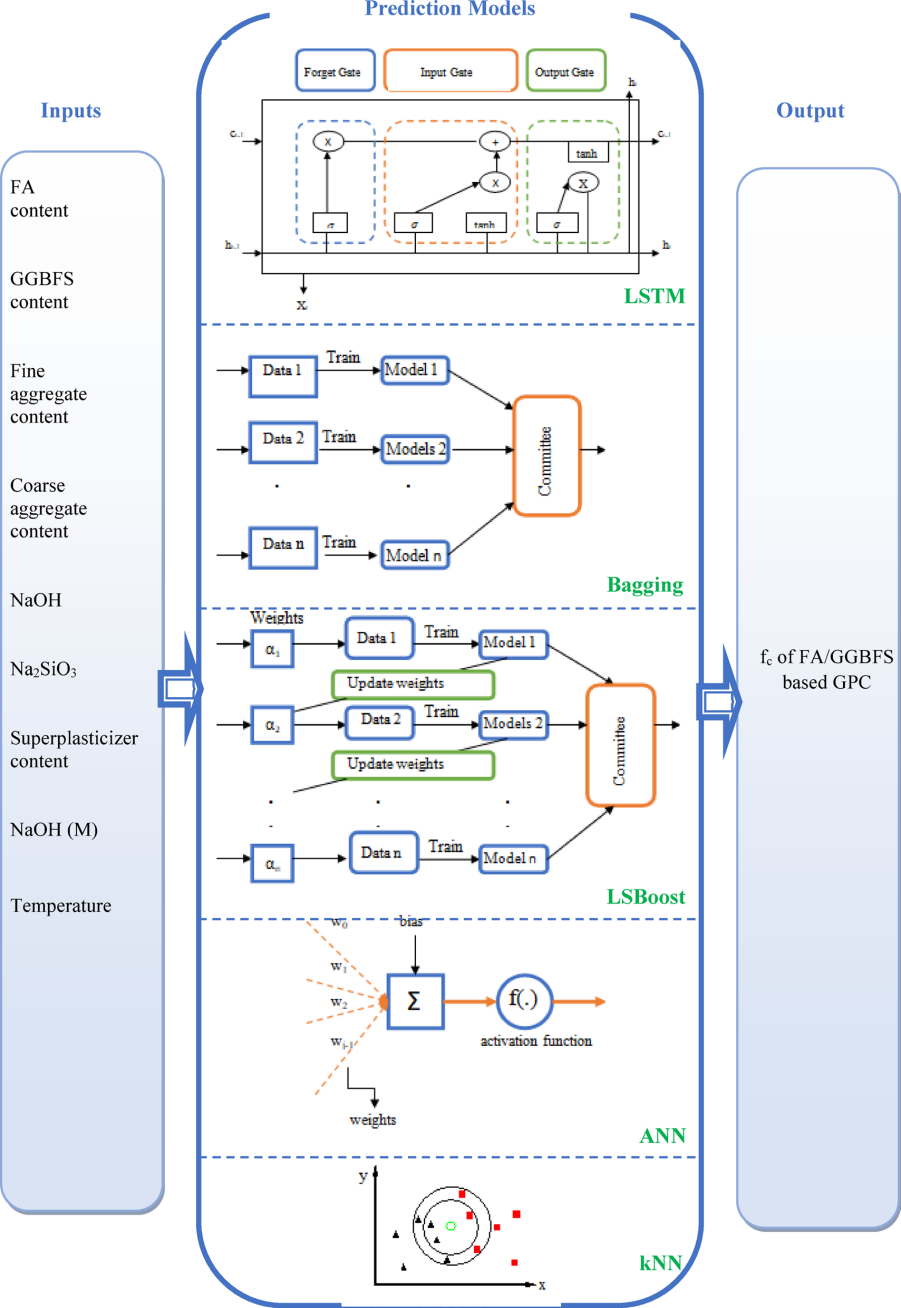
Graphical representations of input variables and model architectures used for f_c_ prediction.

The data were randomly divided into training and testing groups. In the training dataset, 90% of the total data were utilized to train the model. Then, the performance of the proposed model was evaluated through the testing dataset, which was the remaining (10%) data. Many studies in the literature^40,74–77^ used the split ratio of 90% and 10% due to achieving better results which could be explained by training the models on a higher number of observations. Five different prediction models, namely LSTM, Bagging, LSBoost, ANN, and kNN, were developed to forecast the f_c_ of FA/GGBFS-based GPC samples. Furthermore, the performances of these developed models were evaluated via six different statistical parameters. The flowchart of the current study was summarized in Fig. 1, and the graphical representation of the data-driven models was given in Fig. 4.

### Sensitivity analysis

Sensitivity analysis (SA) is a simple method to determine the influence of each input parameter on the output parameter. It provides feedback on which parameters are valid, and therefore, by removing the unimportant ones, the input parameters will be reduced, and then the model complexity and the required time for training will also be decreased. It is possible that the consequences of SA help researchers remove some input parameters, and thus, better analysis is achieved with higher performance prediction.

In the current study, the cosine amplitude method is one of the most successful and common techniques^40,78–80^was utilized to perform SA. A collection of data samples is used to construct a data array, $\:X$, as follows:

2 $$\:X=\:\left\{{x}_{1},\:{x}_{2},\:{x}_{3},\:\dots\:\dots\:,\:{x}_{n}\right\}$$

Each of $\:{x}_{i}$ in the array $\:X$, is a vector of length m:

3 $$\:{x}_{i}=\:\left\{{x}_{i1},\:{x}_{i2},\:{x}_{i3},\:\dots\:\dots\:,\:{x}_{im}\right\}$$

The relation between the dataset of $\:{X}_{i}$ and $\:{X}_{j}$. $\:{R}_{ij}$ is expressed as follows^81^:

4 $$\:{R}_{ij}=\:\frac{\sum\:_{k=1}^{m}{x}_{ik}{x}_{jk}}{\sqrt{\sum\:_{k=1}^{m}{x}_{ik}^{2}\sum\:_{k=1}^{m}{x}_{ik}^{2}}}$$

## Development of data-driven models

### Long short-term memory (LSTM)

LSTM is a powerful technique implemented by Hochreiter and Schmidhuber^82^ to overcome the traditional Recurrent Neural Networks (RNNs) limitations when encountering long-term dependencies in sequential data. Traditional RNNs face obstacles such as disappearance or bursty gradient problems, which weakens their capacity to effectively assimilate and disseminate information over long sequences. LSTMs overcome this challenge by introducing a more complex structure consisting of special gates and memory cells. Memory cells are storages that preserve information over time. There are three gates, including the input, forget, and output gates, which regulate the information flow into, out of, and within the memory cells, as shown in Fig. 4. This mechanism allows LSTMs to update and retain relevant information while discarding unnecessary data^82^.

5 $$\:{f}_{t}=\:\sigma\:\:({U}_{g}{x}_{t}+\:{W}_{g}{h}_{t-1}+\:{b}_{f})$$

6 $$\:{i}_{t}=\:\sigma\:\:({U}_{i}{x}_{i}+\:{W}_{i}{h}_{t-1}+\:{b}_{i})$$

7 $$\:{\mathop {\text{m}}\limits^{\prime } }_{t}=\:tanh\:({U}_{m}{x}_{t}+\:{W}_{m}{h}_{t-1}+\:{b}_{m})$$

8 $$\:{m}_{t}=\:{g}_{t}*{m}_{t-1}+\:{i}_{t}*\:{\mathop {\text{m}}\limits^{\prime } }_{t})$$

9 $$\:{o}_{t}=\:\sigma\:\:({U}_{o}{x}_{t}+\:{W}_{g}{h}_{t-1}+\:{b}_{o})$$

10 $$\:{h}_{t}=\:{o}_{t}*\text{t}\text{a}\text{n}\text{h}\left({c}_{t}\right)$$

$\:U$ and $\:W$ are input weights in the gates of input ($\:{i}_{t})$, modulate input ($\:{\mathop {\text{m}}\limits^{\prime } }_{t})$, forget $\:{(f}_{t})$, and output ($\:{o}_{t})$. $\:{h}_{t}$, $\:{c}_{t}$, and $\:b$ are a hidden condition, cell state, and bias function, respectively. Two controllers decide how much information should be taken from the last loop and how much information should be transferred to the new state. As the distance between loops increases, RNN may lose its ability to link information. However, LSTMs have shown remarkable effectiveness in capturing long-term dependencies within sequential data due to the unique nature of its repeat module^83^. A notable success has been achieved by LSTM in the fields of speech recognition, natural language processing, and sentiment analysis^82^.

### Bootstrap aggregating (bagging)

Bagging is an ensemble algorithm that addresses the distribution of the prediction model by adding extra training data. There are two steps, which are bagging and aggregating. The training dataset is divided for each weak learner via random sampling in bagging, and it involves replacement of data from the original set. It shows the distribution of data and causes adverse bias, which helps to develop a stronger model. Certain observations can be repeated for each new training dataset when sampling is done using replacement. After the procedure of bagging, each element has the same probability of occurrence in the newly created dataset. In the aggregating part, based on the training dataset, multiple random subsets are formed, and their mean values are calculated. Then, the average of these mean values is used as the new predicted mean of the samples. After that, aggregating is applied for the final prediction, which can be defined as follows^84^:

11 $$\:{f}_{bagging}=\frac{1}{B}+\sum\:_{m=1}^{B}{f}^{+b}\left(x\right)$$

where, $\:{f}^{+b}\left(x\right)$ is the result obtained from b-th training dataset, and $\:B$ is the total number of the bootstrapped training dataset. The non-overfitting prediction error is reduced by using a sufficiently large number of bootstrapped training dataset. Besides, fluctuation can be significantly reduced by adjusting the forecast so that it corresponds to the desired result^85^. The Bagging model structure was shown in Fig. 4.

**Fig. 5:**
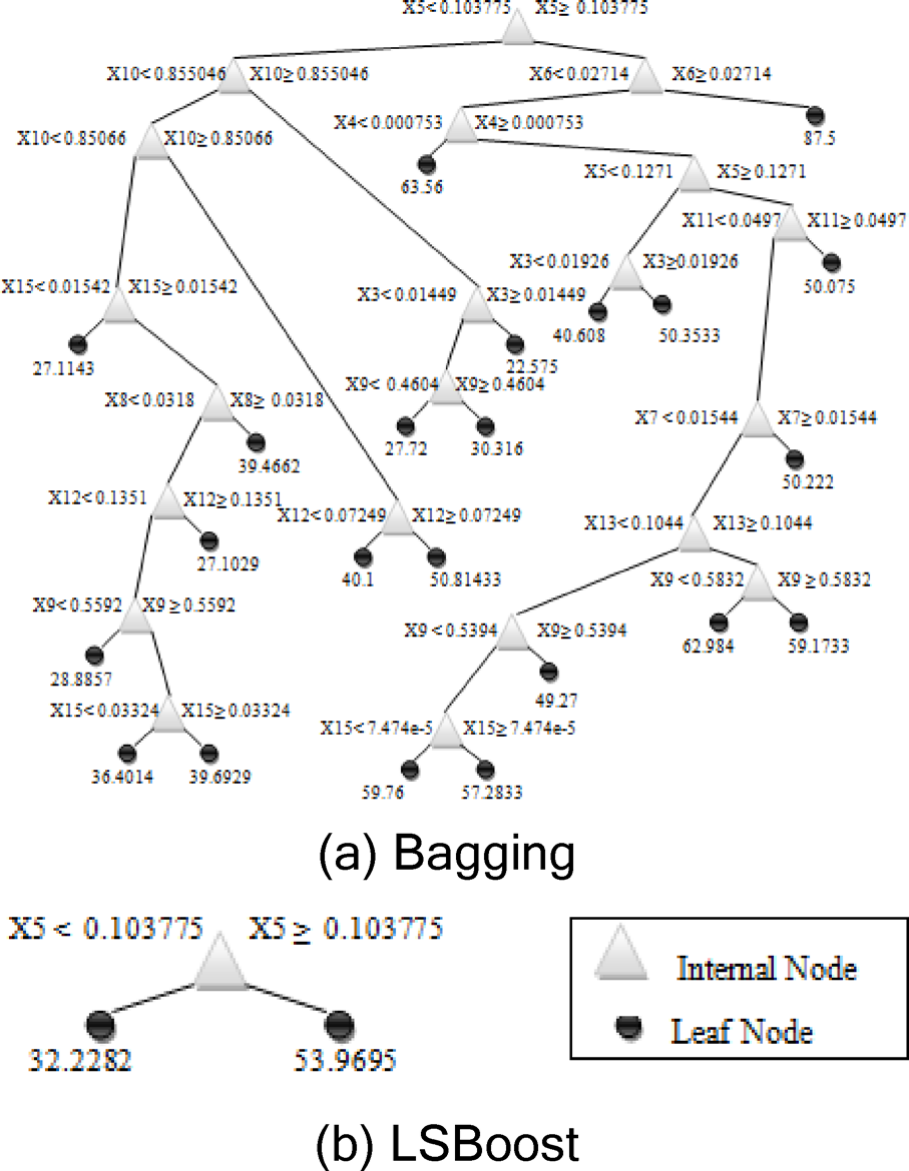
The diagrams of the Bagging and LSBoost models for the f_c_ prediction.

In the current study, as given in Fig. 5(a), the devised Bagging model is characterized by 43 nodes.

### Least-squares boosting (LSBoost)

LSBoost is a type of ensemble learning algorithm proposed by Valiant and Kearns^86^ by combining several weak supervised models with the goal of obtaining a more comprehensive, powerful supervised model. Unlike the Bagging model, LSBoost algorithms adopt a strategy to decrease the bias of the previous round learning process and consequently, it can simplify each basis regressor to reduce variance based on ensuring bias. A balance between variance and bias is necessary to develop an ideal prediction model. In the process of the generation of the algorithm, let the training dataset is $\:D=\:\left\{\left({x}_{1},\:{y}_{1}\right),\:\left({x}_{2},\:{y}_{2}\right),\dots\:.,\:\left({x}_{n},\:{y}_{n}\right)\right\}$ where $\:{x}_{i}$ ∈ **X** ⊂ $\:\:{R}^{n}$, $\:{y}_{i}$ ∈ **Y** ⊂ $\:\:R$, i = 1, 2,., N. The optimal variables $\:i$ and s are solved as follows;

12 $$\:\underset{j,x}{\text{min}}\left[\underset{{c}_{1}}{\text{min}}\sum\:_{{x}_{i}{\text{R}}_{1}\left(\text{j},\text{s}\right)}{\left({y}_{i}-{c}_{1}\right)}^{2}\:+\:\underset{{c}_{2}}{\text{min}}\sum\:_{{x}_{i}{\text{R}}_{2}\left(\text{j},\text{s}\right)}{\left({y}_{i}-{c}_{2}\right)}^{2}\right]$$

13 $$\:{R}_{1}\left(j,\:s\right)=\:\left\{x|{x}^{\left(j\right)}\le\:s\right\}$$

14 $$\:{R}_{2}\left(j,\:s\right)=\:\left\{x|{x}^{\left(j\right)}>s\right\}$$

15 $$\:{ \widehat{{\text{c}}} }_{m}=\:\frac{1}{{N}_{m}}\:\sum\:_{{x}_{i}{\text{R}}_{\text{m}}\left(\text{j},\text{s}\right)}{y}_{i},\:x{R}_{m},\:m=1,\:2$$

These steps given in Eqs. 12–15 continue until the stop condition is met. Then, the input space is divided into M regions to form the regression tree:

16 $$\:f\left(x\right)=\:\sum\:_{m=1}^{M}{\widehat{{\text{c}}}}_{m}I\:(x{R}_{m})$$

in which $\:N$ is the sample numbers, $\:{R}^{n\:}$is the n sets of real numbers, $\:c$ is the output variable and, $\:{\widehat{{\text{c}}}}_{m}$is the estimate of output^86^. The structure of the LSBoost model was given in Fig. 4. In this study, the devised LSBoost model is characterized by three nodes as given in Fig. 5(b).

### Artificial neural network (ANN)

ANNs are nonlinear statistical data modeling tools for relationships between input and output data. It is a network that changes its structure according to the information flowing through it during the phase of learning^87^. The input layer consists of input parameters and the same number of input neurons, while the output layer includes the results and the same number of output neurons. The hidden layer is between the input and output layers. In ANN model, there is a loop from the output of the hidden layer to the input layer. The final model result is affected by several parameters, such as the number of neurons, hidden layer, training algorithm, and transfer function^88^.

One of the benefits of applying neural networks is that easier to use and more accurate models of complex natural systems having large inputs^89^. Neural networks have simple operating elements that work in parallel. The performance of these systems depends on the interconnections within the components and inspired by the human brain^90^. Therefore, it is possible to develop an artificial structure suitable for natural networks and decide the relation between its components by adjusting the value of each link according to its weight. After training, applying a specific input into the network leads to a particular output. Minimizing error is the most important part of training. It can be achieved by changing the weight in the process of learning and continuing this till the function of error becomes less than the specified limit. The error is as follows:

17 $$\:Error=\:\frac{1}{N}\:\sum\:_{i=1}^{N}{\left({Y}_{p}-{Y}_{m}\right)}^{2}\:$$

In where, $\:N$, $\:{Y}_{p}$, and $\:{Y}_{m}$ are the number of samples, target, and predicted results, respectively. It is an iterative procedure and the error is high in the initial step due to selecting the weights randomly. Since estimating the weights by trial and error would require a lot of effort and time, the gradient descent approach using the error gradient to reduce the error can be an effective method^91^. This method is called backpropagation, which includes backward and forward phases during training. Signals travel towards the output node, and errors are evaluated for each node. The network corrects the bias, inputs and layer weights in the backward stage^92^. The calculation of ANN output with one node can be formulated as follows:

18 $$\:Output=\:\frac{{w}_{N1}}{{1+e}^{-\beta\:}}+\:threshold$$

19 $$\:\beta\:=bias+\:\sum\:_{i=1}^{p}\left({x}_{i}\:x\:{w}_{i}\right)$$

In Eqs. 18 and 19, $\:{w}_{N1}$, and threshold are the input weight from node 1 and the error of the output layer, respectively. $\:bias$, $\:p$, $\:{x}_{i}$, and $\:{w}_{i}$ are the error of the hidden layer, independent variable numbers, predictors value, and their corresponding weights, as shown in Fig. 4.

### K-Nearest-Neighbours (kNN)

kNN is first developed by Altman^93^ for classification problems. In the algorithm, all observations are stored at first, and then, the other observations are estimated on the basis of distance functions. It basically aims to evaluate a numerical target averaged from the k’s nearest neighbors^94^. Besides, the inverse distance weighted average is used to calculate this distance^95^. The distance functions in regression-related problems used by kNN to evaluate the distance between neighbors as follows:

20 $$\:\text{M}\text{a}\text{n}\text{h}\text{a}\text{t}\text{t}\text{a}\text{n}\:\text{f}\text{u}\text{n}\text{c}\text{t}\text{i}\text{o}\text{n}\::\:\:\:\:\sum\:_{i=1}^{f}\left|{x}_{i}-{y}_{i}\right|$$

21 $$\:\text{E}\text{u}\text{c}\text{l}\text{i}\text{d}\text{e}\text{a}\text{n}\:\text{f}\text{u}\text{n}\text{c}\text{t}\text{i}\text{o}\text{n}:\:\sqrt{\sum\:_{i=1}^{f}{\left({x}_{i}-{y}_{i}\right)}^{2}}$$

22 $$\:\text{M}\text{i}\text{n}\text{k}\text{o}\text{w}\text{s}\text{k}\text{i}\:\text{f}\text{u}\text{n}\text{c}\text{t}\text{i}\text{o}\text{n}:\:{\left(\sum\:_{i=1}^{f}{\left(\left|{x}_{i}-{y}_{i}\right|\right)}^{2}\right)}^{1/q}$$

In Eqs. 20–22, $\:{x}_{i}$ and $\:{y}_{i}$ are the i-th dimensions of the $\:x$ and $\:y$ points, respectively, and $\:q$ is the order between $\:x$ and $\:y$ points. The structure of the kNN model was shown in Fig. 4.

## Model implementation and verification

The statistical parameters evaluated to assess the performance of the developed models in the current study were expressed in Eqs. (23–28). These are the most commonly used evaluation metrics^33,45,58,96,97^. In these equations, $\:n$ represents the total number of datasets, $\:{y}_{p}$ and $\:{y}_{e}$ are the predicted and experimental values, respectively. σ is the standard deviation, and $\:m$ is the maximum fluctuation in the input data type.

23 $$\:\text{C}\text{o}\text{e}\text{f}\text{f}\text{i}\text{c}\text{i}\text{e}\text{n}\text{t}\:\text{o}\text{f}\:\text{d}\text{e}\text{t}\text{e}\text{r}\text{m}\text{i}\text{n}\text{a}\text{t}\text{i}\text{o}\text{n}:\:{R}^{2}=1-\:\frac{\sum\:_{i=1}^{n}{\left({y}_{p}-\:{y}_{e}\right)}^{2}}{\sum\:_{i=1}^{n}{\left({y}_{e}-\:{y}_{p}\right)}^{2}}$$

24 $$\:\text{M}\text{e}\text{a}\text{n}\:\text{s}\text{q}\text{u}\text{a}\text{r}\text{e}\:\text{e}\text{r}\text{r}\text{o}\text{r}:\:MSE=\frac{\sum\:_{i=1}^{n}{\left({y}_{p}-\:{y}_{e}\right)}^{2}}{n}$$

25 $$\:\text{R}\text{o}\text{o}\text{t}\:\text{m}\text{e}\text{a}\text{n}\:\text{s}\text{q}\text{u}\text{a}\text{r}\text{e}\:\text{e}\text{r}\text{r}\text{o}\text{r}:\:RMSE=\sqrt{\frac{\sum\:_{i=1}^{n}{\left({y}_{p}-\:{y}_{e}\right)}^{2}}{n}}$$

26 $$\:\text{N}\text{o}\text{r}\text{m}\text{a}\text{l}\text{i}\text{z}\text{e}\text{d}\:\text{r}\text{o}\text{o}\text{t}\:\text{m}\text{e}\text{a}\text{n}\:\text{s}\text{q}\text{u}\text{a}\text{r}\text{e}\:\text{e}\text{r}\text{r}\text{o}\text{r}:\:NRMSE=\frac{RMSE}{}$$

27 $$\:\text{M}\text{e}\text{a}\text{n}\:\text{a}\text{b}\text{s}\text{o}\text{l}\text{u}\text{t}\text{e}\:\text{p}\text{e}\text{r}\text{c}\text{e}\text{n}\text{t}\text{a}\text{g}\text{e}\:\text{e}\text{r}\text{r}\text{o}\text{r}:\:MAPE=\frac{1}{n}\left(\sum\:_{i=1}^{n}\:\left|\frac{{y}_{p}-\:{y}_{e}}{{y}_{p}}\right|\:\right)*\:100$$

28 $$\:\text{P}\text{e}\text{a}\text{k}\:\text{s}\text{i}\text{g}\text{n}\text{a}\text{l}-\text{t}\text{o}-\text{n}\text{o}\text{i}\text{s}\text{e}\:\text{r}\text{a}\text{t}\text{i}\text{o}:\:PSNR=10\:{log}_{10}\frac{{m}^{2}}{MSE}$$

Among these statistical parameters, $\:{R}^{2}$ is the most commonly used one to evaluate the effectiveness of the devised models. It evaluates the extent to which each independent variable is interpreted relative to changes in the dependent variable and is used to assess how close it is to predicted values. MSE and RMSE are statistical tools used to calculate the difference between actual and predicted values. NRMSE measures this difference relative to a mode. MAPE quantifies the average relative distance between target and output values. PSNR compares the maximum possible power of a signal to the power of the noise that affects its quality. The higher $\:{R}^{2}$ and PSNR values represent more accurate prediction results between the predicted and experimental values, while lower MAPE and RMSE values represent a better fit.

In addition to these performance metrics, a new engineering index, a20-index, has been recently proposed and used^9,98–101^ for the reliability assessment of the devised models. This index is calculated as follows:

29 $$\:a20-index=\:\frac{m20}{M}$$.

In Eq. 29, $\:\text{m}20$ is the sample number with the value of experimental value/predicted value in the range of 0.80 and 1.20, and $\:M$ is the number of dataset samples. This index indicates the sample quantity meeting the predicted values with a deviation of ±20 with regards to experimental values. Therefore, the a20-index value is expected to be the unit value for a perfect prediction model.

In the current study, scatter plots and Taylor diagrams were employed as graphical verification methods for the training and testing phases of the five proposed models. In order to evaluate the proposed models, three parameters of the correlation were compared simultaneously with the Taylor Diagram via RMSE, correlation coefficient, and standard deviation.

## Results

### Sensitivity analysis results

**Fig. 6:**
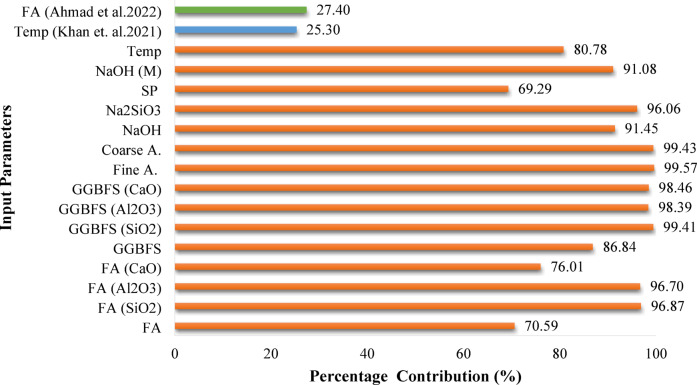
Contributions of input parameters on the output.

The impact of each input variable on f_c_ results of FA/GGBFS-based GPC determined through Sensitivity analysis was shown in Fig. 6. This analysis revealed that fine and coarse aggregate contents had the highest contribution of 99.57% and 99.43%, respectively. The high volume fraction of GPC composite was consisting of aggregate. Therefore, aggregate content affects the mechanical properties of GPC obviously. In the literature, Alanazi^102^ found that the stiffness of aggregate had significant impact on the strength of GPC. However, considering the chemical composition contents of the binders such as SiO_2_, Al_2_O_3,_ and CaO, it was determined that the contributions of GGBFS were quite high with 99.41%, 98.39%, and 98.46, respectively. As for FA, these values were obtained as 96.87%, 96.70 and 76.01% respectively. Besides, Na_2_SiO_3_ content had a contribution of 96.06%, followed by NaOH content (91.45%), NaOH (M) (91.08%), and curing temperature (80.78%). The SP content seems to have the lowest impact on f_c_ with 69.29%. In the study of Ahmad et al.^60^FA provided the highest contribution, followed by NaOH molarity and curing age to forecast the f_c_ of FA-based GPC. Khan et al.^103^ found that curing temperature is the prompting parameter to control the f_c_ of GPC with FA. The highest parameters affected the compressive strength results of GPC found in the studies of Ahmad et al.^60^ and Khan et al.^103^ were also shown in Fig. 6.

**Fig. 7:**
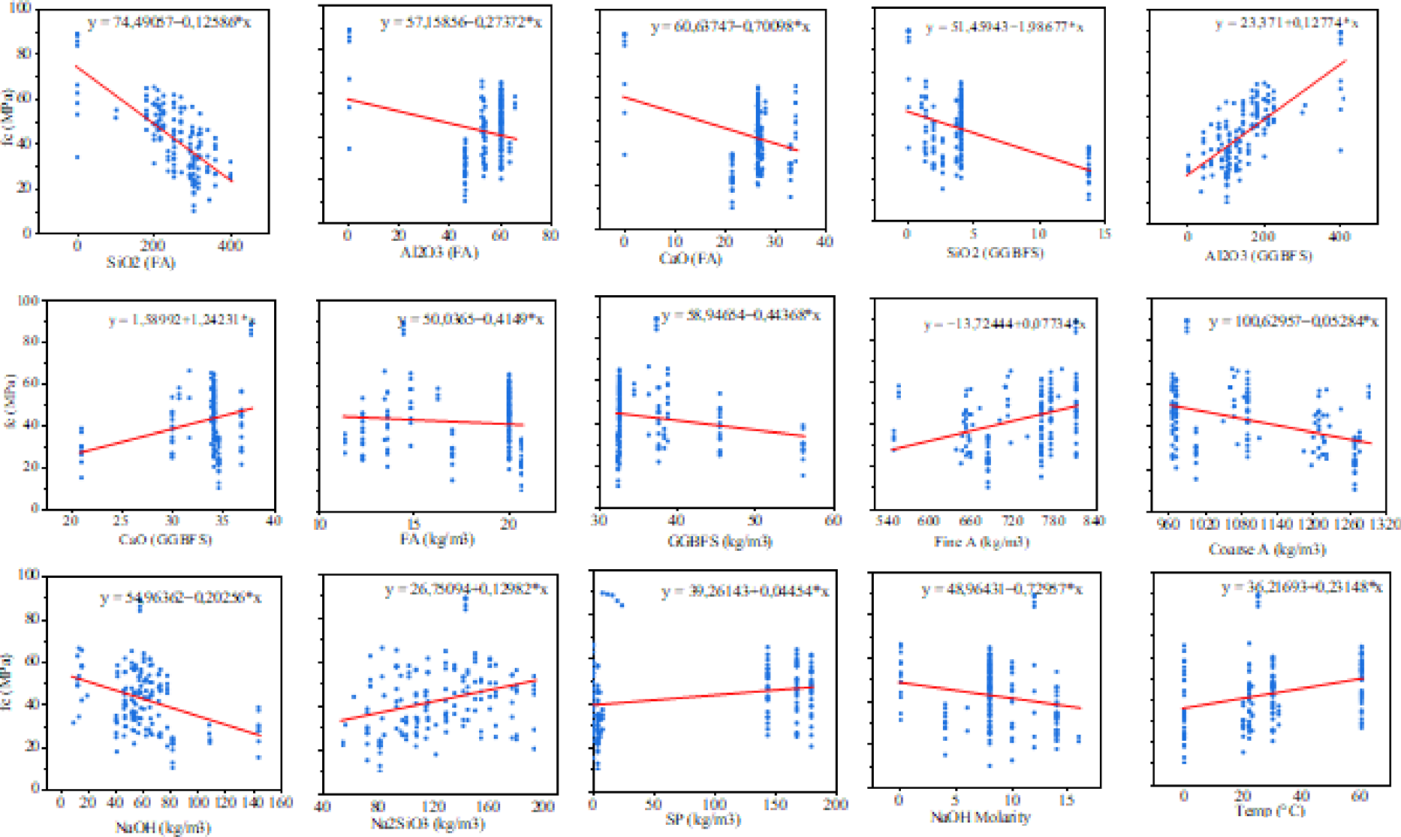
Variation of f_c_ with input parameters.

The variations in the f_c_ with respect to different input parameters were illustrated in Fig. 7. These graphs not only display the data point distributions but also feature a linear regression line representing the relationship between the f_c_ and each input variable. Figure 6 clearly shows that the contents of SiO_2_, Al_2_O_3_, and CaO in the precursors are key factors influencing the strength gain of the GPC, as indicated by the higher slope of the linear regression line. It is evident that the chemical composition of FA and GGBFS significantly influences the properties of geopolymer composites. This can be attributed to their role in the formation of a greater amount of geopolymeric gel. Their presence enhances the formation of a robust geopolymer network, which leads to improved interparticle bonding^21^. The analysis of the variation in f_c_ with respect to the amount of alkali activators revealed that these components significantly contribute to the gain in f_c_. A higher concentration of alkali activators result in increased dissolution of aluminosilicates in a highly alkaline environment, which accelerates the polymerization process and creates a denser internal structure. The addition of more Na_2_SiO_3_ and NaOH enhances the ratio of silicon dioxide to aluminum oxide. This increase in silicon-oxygen-silicon bonds results in greater compressive strength^22^. However, if the concentration of NaOH exceeds a certain level, a reduction in f_c_ occurs. This decline is due to decreased water-gel workability in the matrix, which prevents proper binding of the coarse and fine aggregates^23^. Similar findings were also found in the previous studies^24,25^. The analysis of the variation in the f_c_ concerning curing temperature also highlighted its significance in the strength gain of GPC. Additional heat is essential to accelerate the reaction process, leading to an improved f_c_ of GPC. At higher curing temperatures, moisture from the GPC tends to evaporate, even when the material is properly sealed, as noted in previous literature^26^.

### Hyperparameters of the training process

Hyperparameters have a significant influence on the training process of models due to controlling the learning phase. Model accuracy and training time depend on the optimization of hyperparameters. The hyperparameter values are set before running the training, and these values do not change until the training process finishes. In this study, hyperparameters were tuned by optimization to provide a successful model that meets the desired metric. The number of 139 samples was used as training, and the remaining 16 were selected as testing for all proposed models to predict the f_c_ of FA/GGBFS-based GPC.

**Fig. 8:**
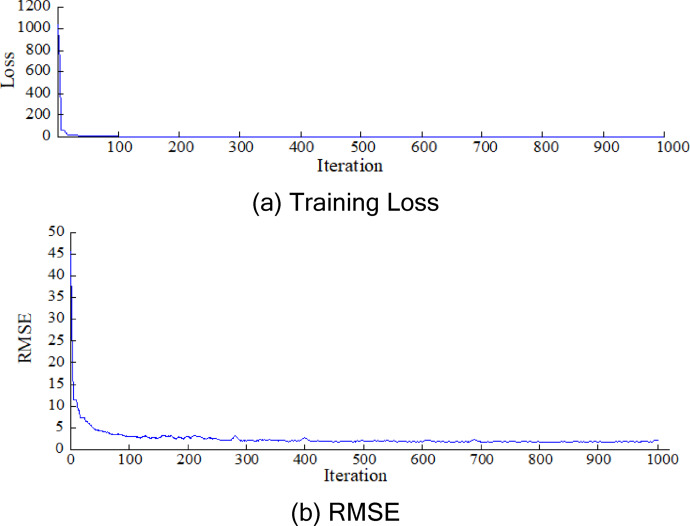
The training process of the LSTM model.

The program used to run the network models was developed using MATLAB software. In the LSTM model, the training method of ‘Adam’ was used after testing optimizing algorithms to achieve the best optimizer. The initial learning rate was 0.15, and the gradient threshold was used as 1. The mini-batch size was set to 100. If the learning rate used for training is too low, the training process takes a long time. Conversely, if learning rate is too high, the training may result in suboptimal performance. To manage the learning rate, it was adjusted using a drop factor of 0.000001 during training, which spanned 1000 epochs. This drop factor is a multiplicative value applied to the learning rate each time a specific number of epochs is completed. The dropout algorithm was implemented to prevent overfitting in the LSTM model during training. The training process of the LSTM model was given in Fig. 8. The training loss and RMSE values are quite high, indicating poor performance when the training starts. However, after enough iteration, both the RMSE and training loss decrease to a relatively low level, which signifies an acceptable outcome.

The individual trees of ensemble Bagging and LSBoost models were given in Fig. 5 to show the model specifications. In the visualization, black circles are the node prediction, and triangles are the splitting rules. The ‘fitensemble’ function was utilized to run the Bagging and LSBoost algorithms in the MATLAB software. The ‘Bag’ learner in MATLAB was employed for Bagging, while ‘LS-Boost’ was used to combine weak learners and create an accurate ensemble for the LSBoost model. The optimal values of the hyperparameters of these models were determined by Bayesian optimization. In the LSBoost model, the results indicated that lower learning rates required more cycles to reach optimal performance, while higher learning rates allowed for faster convergence but resulted in greater variability in model performance. The best outcomes were achieved with lower learning rates paired with a moderate number of cycles. The learning rate of 0.1 and 100 learning cycles were used in LSBoost model. For the kNN model, the developed algorithm first calculated the euclidean distance and then counted the data points in each category among K neighbors. New data points were assigned to the category with the most neighbors. The algorithm and weight function were set as ‘auto’ and ‘uniform’, respectively. The number of neighbors was used as 7.

**Fig. 9:**
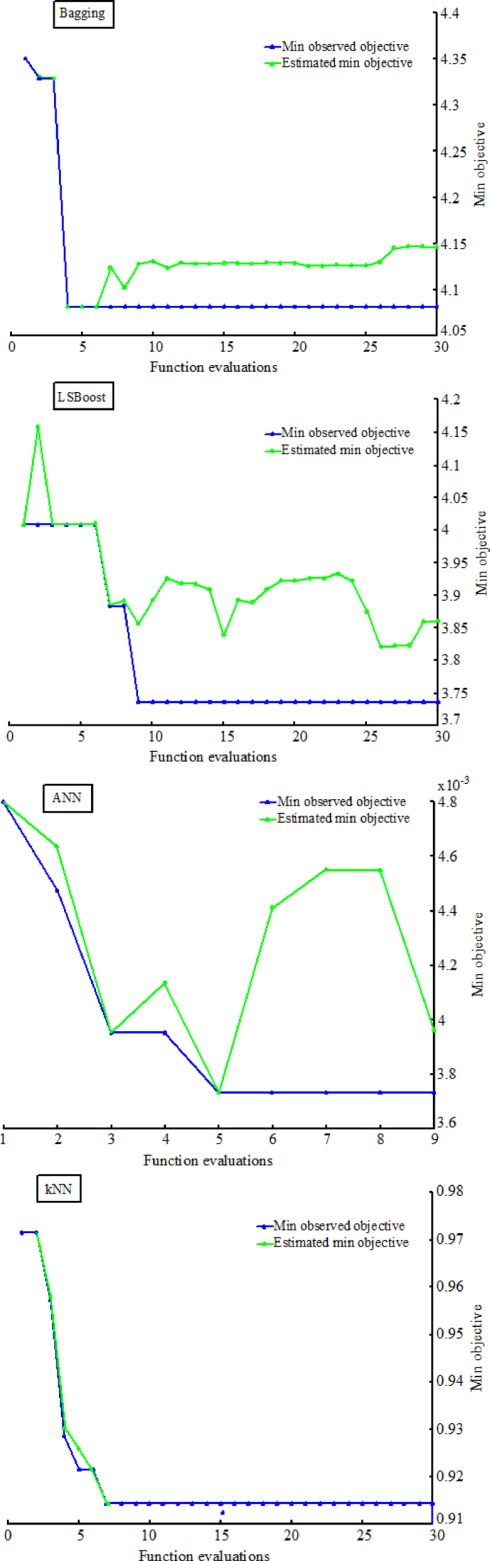
The hyperparameter optimization of proposed models.

After optimizing the hyperparameters of Bagging, LSBoost, ANN, and kNN models, their training and testing performance were illustrated in Fig. 9. The green lines indicate the estimated minimum objective value at each iteration, reflecting the algorithm’s understanding of the optimum and the blue lines represent the actual observed minimum value of the objective function, showcasing the performance achieved during the optimization process. This plot illustrates the gradual improvement of optimization as the algorithm enhances its understanding of the problem, aiming to optimize the objective function value through iterative exploration and exploitation.

In ANN model development, the multi-feed-forward network model with one hidden layer was utilized. The accuracy of the ANN model depends on determining the optimal number of hidden layer neurons. However, there is a lack of theory regarding finding the optimal value. Therefore, after trying two neurons, it was increased by adding one neuron. The performance of ANN was monitored for each hidden neuron number, and it continued until the best performance was achieved with an acceptably small error. The best performance was obtained from the ANN model with 20 hidden-layer neurons. Due to obtaining the most appropriate training function, the gradient descent algorithm with moment back-propagation was utilized for training. To prevent overfitting, the Bayesian regularization technique was used during training. While its training speed is slower than other regularization methods, it was chosen for its effectiveness against overfitting, especially with limited datasets.

### Performance of the models

The outcomes obtained from the developed algorithms, namely LSTM, Bagging, LSBoost, ANN, and kNN, in terms of statistical parameters for each training and testing phase, were presented in Table 3. The values of the performance parameters are lower between the training and testing datasets, indicating less probability of overfitting.

The R-squared correlation coefficient and PSNR values of ANN were the highest with 0.9912 and 45.3043, respectively, while the values of MSE, RMSE, NRMSE, and MAPE were the lowest with 1.9171, 1.3846, 0.0175 and 2.1153, respectively, for the training dataset. Besides, a20-index was 0.96. LSBoost model also had higher R-squared and PSNR values with 0.9889 and 44.2708, respectively, and it had lower MSE, RMSE, NRMSE, and MAPE values with 2.4322, 1.5596, 0.0197, and 3.4071, respectively, compared to other proposed models, for the training dataset. The A-20 index of the LSBoost model was also 0.96 as in ANN. Moreover, the performance metrics of LSTM were 0.9679, 7.8221, 2.7968, 0.0354, 5.7041, 39.1976, and 0.94 for R-squared, MSE, RMSE, NRMSE, MAPE PSNR and a-20 index, respectively, for the training dataset. For Bagging, these values were 0.9020, 24.0051, 4.8995, 0.0619, 10.3383, 34.3278, and 0.93, respectively. The worst performance for the training dataset was obtained for the kNN model, which has the values of 0.4341, 214.2003, 14.6356, 0.185, 26.997, 24.8226, and 0.55, respectively.

Considering the testing dataset, the highest values of R-squared, PSNR, and a20-index were obtained with 0.9819, 40.0839, and 1.00, respectively, and the lowest MSE, RMSE, NRMSE, and MAPE values were obtained with 6.3780, 2.5255, 0.0398, 4.5662, respectively, from LSTM model. R-squared, PSNR, and a20-index were also higher for the ANN model with 0.9501, 34.41, and 0.94, respectively, for the testing dataset, while MSE, RMSE, NRMSE, and MAPE values were lower than other models with the values of 23.5549, 4.8533, 0.0764 and 8.9476, respectively. The A-20 index of the testing dataset was 0.94 for the ANN model. For the LSBoost model, R-squared, MSE, RMSE, NRMSE, MAPE PSNR, and a-20 index were 0.9473, 22.7856, 4.7734, 0.0752, 8.9706, 34.5542 and 0.81, respectively. In the case of Bagging, these values were 0.88410, 57.5007, 7.58290, 0.1194, 12.8250, 30.5341 and 0.75, respectively. The worst performance for the testing dataset was also obtained from the kNN model, which has values of 0.5854, 240.0562, 15.4937, 0.244, 26.1872, 24.3277, and 0.31, respectively. The lowest accuracy and poor performance of the kNN model can be explained by its vulnerability to redundant features due to all features being treated equally, despite performing robustly on noisy training sets and training with only one main hyperparameter (k)^104,105^. Kang et al.^4^ reported that the kNN model showed poor performance in predicting the f_c_ of steel fiber-reinforced concrete. Similarly, Asadi et al.^5^ found that kNN model predicted the f_c_ of concrete having waste marble powder with significant discrepancies. These findings suggest that the kNN technique may not be effective in predicting the f_c_ of various concrete types. This could be due to the algorithm treating all characteristics equally, even though these characteristics may have different levels of influence on the f_c_ of concrete^5^.

**Table 3:** Performance metrics of the models.

	Statistical criteria	Models				
		LSTM	Bagging	LSBoost	ANN	kNN
Training	R^2^	0.9679	0.9020	0.9889	0.9912	0.4341
	MSE	7.8221	24.0051	2.4322	1.9171	214.2003
	RMSE	2.7968	4.8995	1.5596	1.3846	14.6356
	NRMSE	0.0354	0.0619	0.0197	0.0175	0.185
	MAPE	5.7041	10.3383	3.4071	2.1153	26.997
	PSNR	39.1976	34.3278	44.2708	45.3043	24.8226
	a20-index	0.94	0.93	0.96	0.96	0.55
Testing	R^2^	0.9810	0.88410	0.9473	0.9501	0.5854
	MSE	6.3780	57.5007	22.7856	23.5549	240.0562
	RMSE	2.5255	7.58290	4.7734	4.8533	15.4937
	NRMSE	0.0398	0.11940	0.0752	0.0764	0.244
	MAPE	4.5662	12.82500	8.9706	8.9476	26.1872
	PSNR	40.0839	30.53410	34.5542	34.41	24.3277
	a20-index	1.00	0.75	0.81	0.94	0.31

**Fig. 10:**
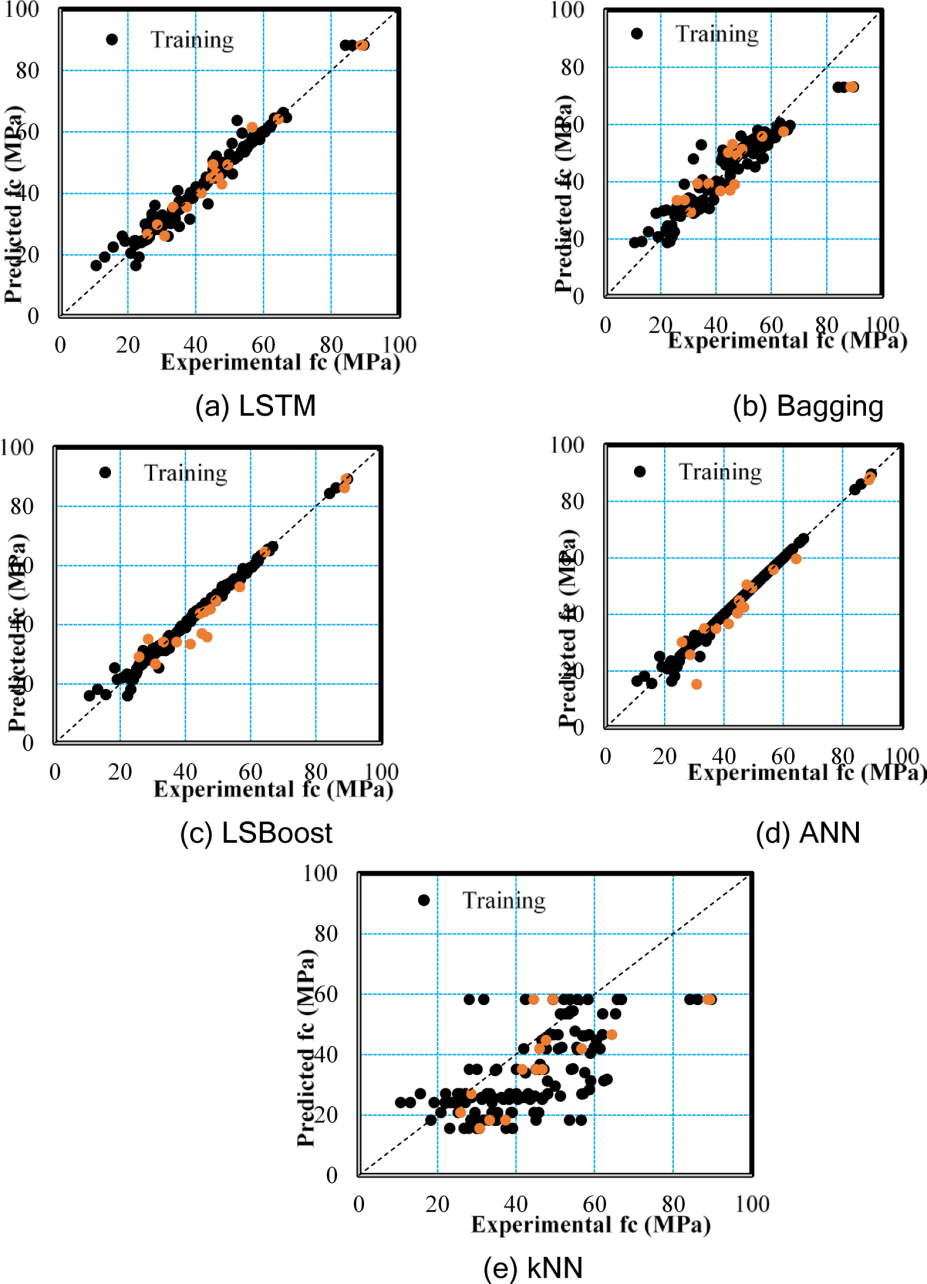
The predicted–experimental values in the training–testing phase of the proposed models.

**Fig. 11:**
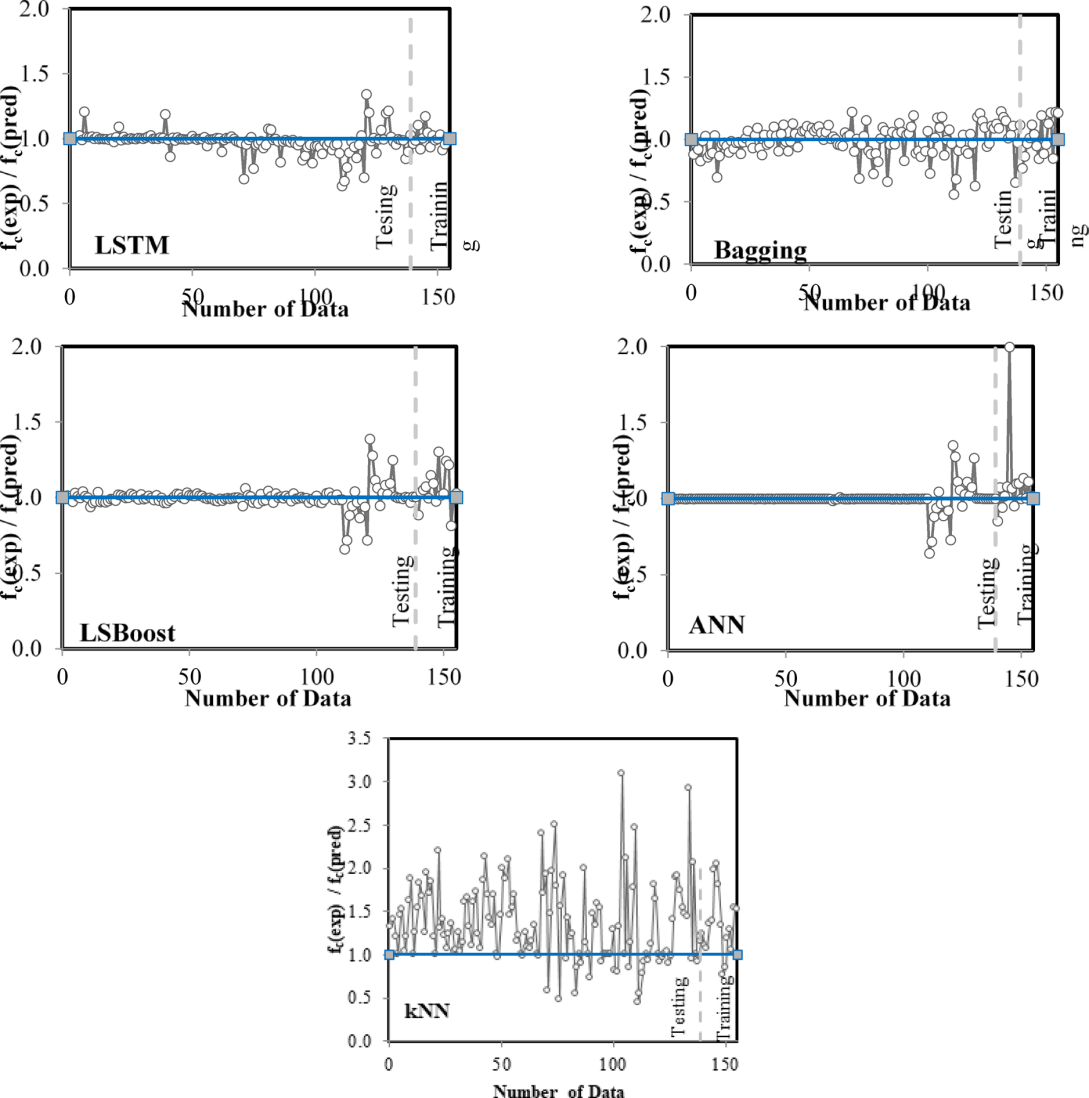
Experimental/predicted f_c_ ratios for the devised models.

The performance of the models was illustrated in Fig. 10 by comparing the actual and predicted f_c_ values for the training and testing dataset. The results indicate that the ANN model, as reflected in the performance metrics, had a training dataset that is closer to the diagonal line than the other models. This suggests a higher accuracy in predictions and a better match between actual and predicted values. However, a scatter plot of the LSTM model demonstrated a higher degree of stability for the training dataset, as the experimental values matched more closely with the predicted ones. In contrast, for the kNN model, the values were scattered throughout the graph, and they are far from the perfect prediction, which shows the lowest accuracy. The same outcome can be inferred from Fig. 11, which shows the ratio of experimental / predicted f_c_ of FA/GGBFS-based GPC. Although the training dataset of ANN was the closest model to 1, in overall, LSTM and LSBoost also showed a strong alignment between experimental and predicted values. Conversely, the results of the Bagging and kNN model indicated that the errors between the experimental and predicted values are higher, especially for the kNN model.

**Fig. 12:**
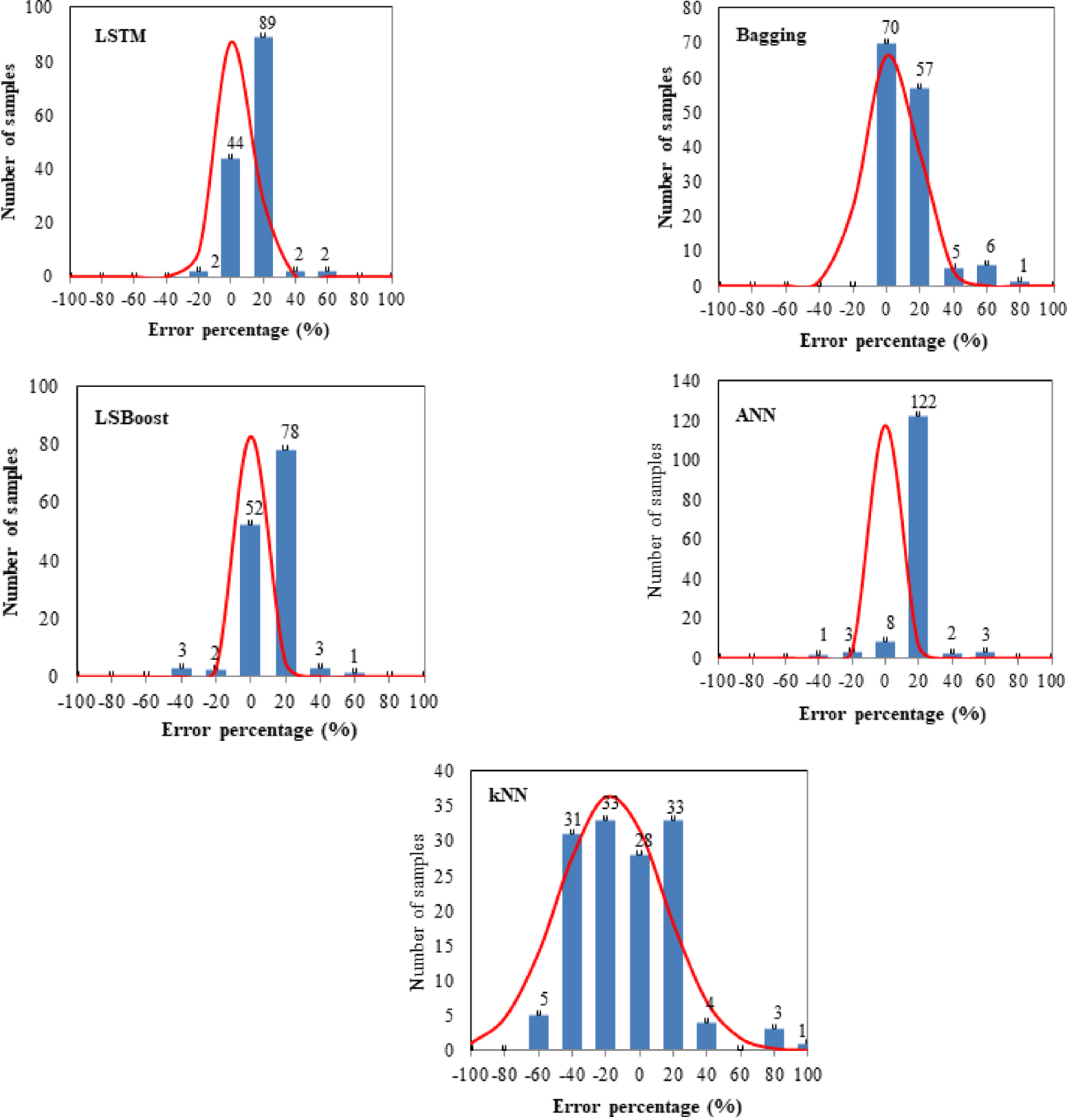
Error percentage distributions and normal distribution fittings of devised models for all dataset.

The error percentage distribution of all datasets, including training and testing, were calculated and illustrated in Fig. 12. It was observed that the error percentage distribution of the proposed models conforms to the normal distribution graph. Except for the kNN model, the error percentages of the samples were close to zero, which can be proven by the high MAPE results of the kNN model (≈ 26). For LSTM, Bagging, LSBoost, ANN, and kNN models, 135, 127, 132, 133, and 94 numbers of samples were within the error percentage range of 20% and − 20%, respectively. The reliability and good applicability of LSTM, Bagging, LSBoost, and ANN algorithms for f_c_ estimation of FA/GGBFS-based GPC were confirmed. Based on all datasets including training and testing, the LSTM algorithm had the highest precision, followed by ANN, LSBoost, and Bagging. However, it is worth noting that the number of samples within the error percentage range of 20% and − 20% was so close to each other for these four prediction models.

**Fig. 13:**
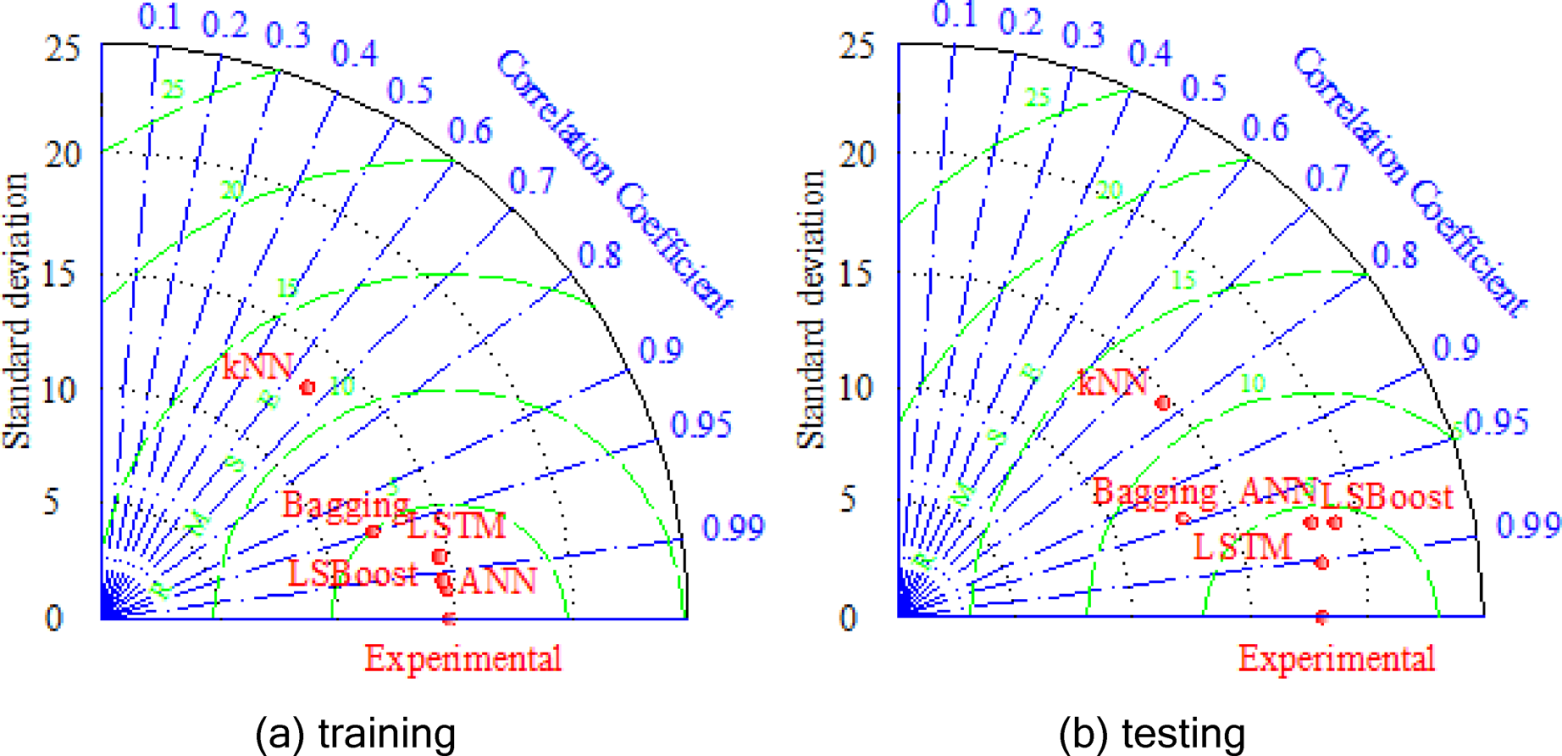
Taylor diagrams.

The Taylor diagram is a valuable tool for comparing the proposed models based on their correlation coefficient, RMSE, and standard deviation. Figure 13 indicated the Taylor diagram for the proposed models using both the training and testing datasets. The point of ‘Experimental’ in Fig. 13 shows the most accurate model, which exhibits the smallest RMSE and the highest correlation coefficient. As can be seen, in the training phase, ANN was the closest model to the ‘Experimental’ point, while in the testing phase, the LSTM model performed best. However, both LSBoost and Bagging models also demonstrated high performance in both phases, especially the LSBoost model, which was notably competitive with ANN and LSTM. On the other hand, the kNN model showed a significantly higher standard deviation, RMSE, and correlation coefficient in the Taylor diagrams for both training and testing phases, highlighting its poorer performance compared to the other models.

### Discussion

**Fig. 14:**
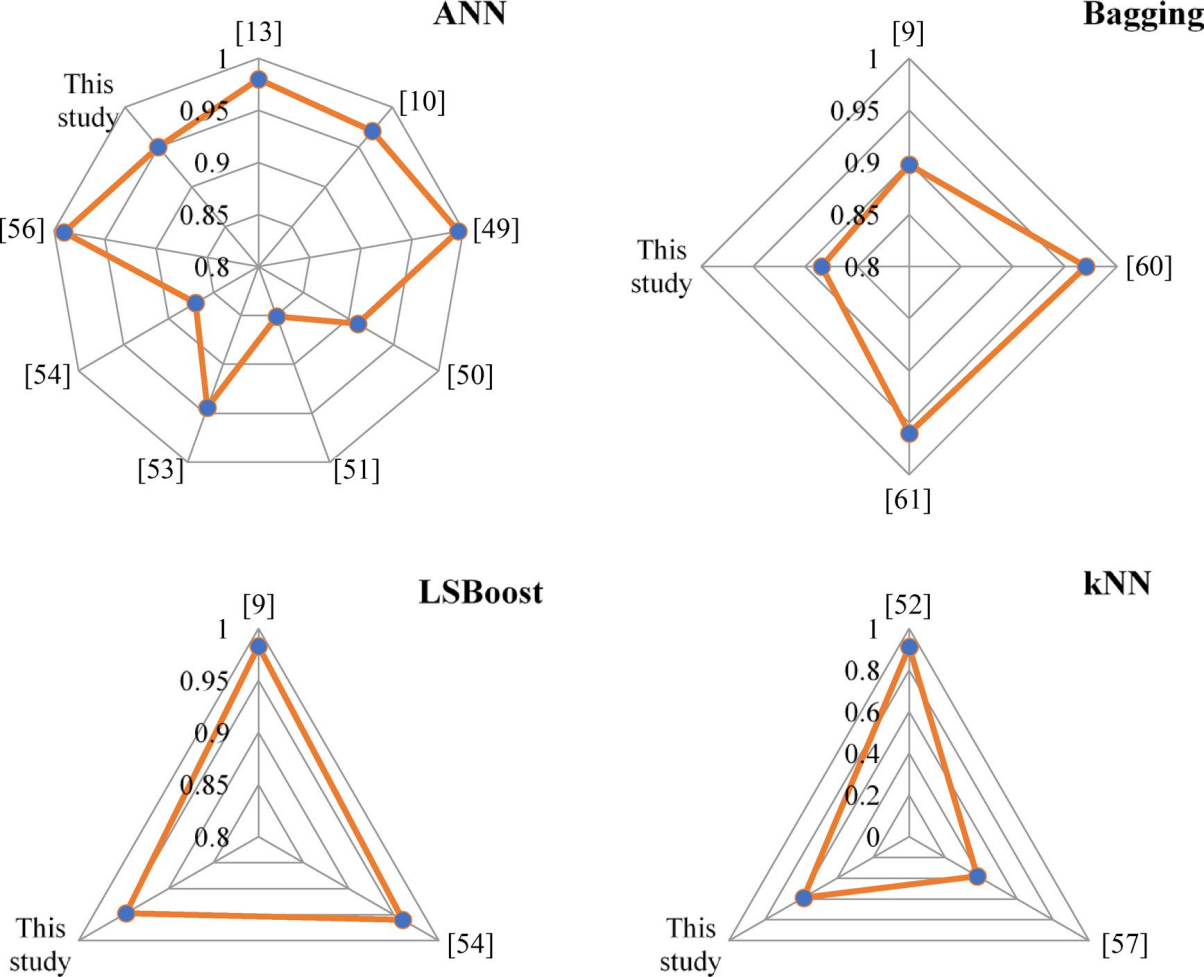
Performance comparison (R^2^ of the devised models in the current study with the previous studies.

Figure 14 shows the R-squared values of the developed algorithms in the current study, namely ANN, Bagging, LSBoost, and kNN, with the previous results obtained in the literature. There has been no any study about the f_c_ prediction of FA/GGBFS-based GPC via the LSTM model, so a graph regarding LSTM has not been prepared.

As mentioned in the section of the introduction and illustrated in Fig. 14, ANN is the most widely used algorithm to predict the f_c_ of GPC blended with FA/GGBFS as the binder. Based on Table 1, the researches about the f_c_ prediction of FA/GGBFS-based GPC via ANN and their results can be summarized as follows;

Shahmansouri et al.^13^ predicted the f_c_ of SF and NZ-based GPC using the input parameters of SF, natural zeolite, GGBFS, age and NaOH concentration via ANN model with R and MSE error values of 0.98 and 4.7769, respectively. In the study of Pazouki^10^the devised ANN model had R^2^ = 0.97 and R^2^ = 0.9 for the training and testing dataset, respectively, to predict the f_c_ of GPC with FA. Their input parameters were FA, fine and coarse aggregate, curing period, temperature, age, water, superplasticizer, molarity, Na_2_SiO_3_ and NaOH content. ANN model developed by Ahmed et al.^49^ predicted the f_c_ of GGBFS/FA-GPC with R^2^ = 0.988 and R^2^ = 0.995 for training and testing datasets, respectively. They selected the input parameters as FA, GGBFS, alkaline solution/binder, fine and coarse aggregate, Na_2_SiO_3_ and NaOH content, SiO2/Al2O3 of FA, SiO2/CaO of GGBFS, Na2SiO3/NaOH and molarity. Nazar et al.^50^ predicted the f_c_ of FA-based GPC with the R-value = 0.91 via ANN by using FA, activator/FA, fine and coarse aggregate, mixing procedure, activator content, water, curing regime and molarity as input parameters. In the study of Dao et al.^51^ANN performed with R^2^ = 0.851 and RMSE = 2.423. Their input parameters were FA, Na_2_SiO_3_ and NaOH content and water. ANN devised by Ling et al.^53^ predicted the f_c_ of FA-based GPC with R^2^ = 0.944. Liquid/FA, alkaline solution concentration, temperature, age and mole ratio were selected as input parameters. In the study of Ahmad et al.^54^the ANN model developed by using the input parameters of FA, Na_2_SiO_3_ and NaOH content, NaOH molarity, curing age, fine and coarse aggregate, SiO_2_ and Na_2_O predicted the f_c_ of GPC having FA with R^2^ = 0.87. The f_c_ of FA/GGBFS-based GPC was predicted by Gupta et al.^56^ with R^2^ = 0.99. They selected the input parameters as FA, superplasticiser, NaOH molarity, rest period, fine and coarse aggregate, NaOH/Na_2_SiO_3_, water and alkaline activator/binder.

Observing Table 1 in the section of Introduction, the ensemble learning approaches, namely Bagging and LSBoost and ML-based algorithm, kNN, have been less concerned for the estimation of f_c_ of FA/GGBFS based GPC. Kina et al.^9^ forecasted the f_c_ of GGBFS-based GPC using the input parameters of GGBFS, SF, natural zeolite, NaOH solution concentration and age via Bagging and LSBoost with R^2^ = 0.898 and R^2^ = 0.983, respectively. In the study of Khan et al.^52^the kNN model predicted the f_c_ of FA-based GPC with R^2^ = 0.911. Their input parameters were FA, fine and coarse aggregate, temperature and curing duration, molarity, alkaline activator/FA, water, Na_2_SiO_3_ and NaOH content. LSBoost model devised by Ahmad et al.^54^ predicted the f_c_ of FA-based GPC with R^2^ = 0.96. They selected the input parameters as FA, Na_2_SiO_3_ and NaOH content, NaOH molarity, curing age, fine and coarse aggregate, SiO_2_ and Na_2_O. Tran^57^ predicted the f_c_ of FA/GGBFS-based GPC using the input parameters of FA, GGBFS, superplasticiser, water, fine and coarse aggregate, Na_2_SiO_3_ and NaOH content, curing period, rest period, curing temperature, NaOH/ Na_2_SiO_3_, alkaline activator/binder and molarity via kNN model with R^2^ = 0.38. In the study of Ahmad et al.^60^the bagging model developed by using the input parameters of FA, fine and coarse aggregate, Na_2_SiO_3_ and NaOH content, NaOH molarity, SiO_2_ and Na_2_O predicted the FA-based GPC with R^2^ = 0.97. Zou et al.^61^ estimated the f_c_ of FA/GGBFS-based using Bagging with R^2^ = 0.96. Their input parameters were FA, GGBFS, Na_2_SiO_3_ and NaOH content, fine and coarse aggregate, NaOH molarity and water/solid.

Considering the literature, the devised ANN models performed with high accuracy for the f_c_ prediction of FA/GGBFS-based GPC. The reliability and high accuracy of the ANN model were also proven in the current study by obtaining better performance metrics for the training dataset, especially. In the training phase, ANN had the highest accuracy with 99.12%, followed by LSBoost and LSTM. It can be emphasized that the accuracy percentages of these three devised algorithms were so close to each other such that LSTM had the R-squared value of 96.8%. Besides, in the testing dataset, deep LSTM outperformed the other ML-based algorithms with 98.1% accuracy, followed by ANN, LSBoost, Bagging, and kNN. As in the case of the training phase, the accuracy percentages of ANN and LSBoost were also close to LSTM, which were 95.0% and 94.73%, respectively. For all datasets, the error percentage distribution showed that the number of samples within the error percentage range of 20% and − 20% were so close to each other for LSTM, ANN, and LSBoost, but it was found that the LSTM algorithm had the highest precision.

As mentioned, the accuracy of the ANN model for the training dataset was the highest, while the deep-LSTM model performed best for the testing dataset as it had the highest accuracy with lower error. In this case, the deep-LSTM can be considered superior compared to other devised models. The ultimate goal of the prediction models is to generalize well to unseen data. The higher accuracy of the testing dataset of Deep LSTM suggests that it generalizes well, even though the accuracy of the training dataset is lower than that of the ANN model. Furthermore, deep-LSTM can be more robust to unseen data due to its higher test accuracy, although its training accuracy is lower than ANN. In general, a model that performs well on unseen data (i.e., the test data) is more desirable.

In addition to the accuracy of the developed model, their complexity is also important for the implementation. Among the developed algorithms, especially LSBoost, ANN and LSTM showed higher performance. Algorithms based on high complexity network for concrete prediction can lead to high computation time and high cost with high complexity network. LSBoost is an ensemble learning algorithm that sequentially combines weak learners to minimize the least squares objective function. Its optimization process is more complex because it fits each new tree to the current ensemble’s residuals, which requires more computational resources and results in increased training time as the number of learners grows. In terms of the ANN model, overfitting and the need for a large number of data in the training process are the main drawbacks^106,107^ and they also increase the complexity of the application. On the other hand, deep learning models such as deep-LSTM are compressed through the network pruning, which effectively suppresses the overfitting and complexity of the neural network without reducing accuracy^108^.

Although researchers confirmed the higher accuracy and the need for minimal training data of the LSTM model, which provides faster predictions in the previous studies ^33,97,109–113^, the LSTM model has not been developed for f_c_ prediction of FA/GGBFS-based GPC before. Therefore, in the current study, the high accuracy of the LSTM model for the prediction of f_c_ of FA/GGBFS-based GPC was proven in terms of performance metrics and error percentage distribution, which show the efficiency of the model devised in this work. Besides, it is also worth to say that LSBoost is one of the best ensemble algorithms for f_c_ prediction of FA/GGBFS-based GPC due to having also high accuracy as proven in the current study and previous works.

## Practical application

The study on predicting the compressive strength of FA/GGBFS-based geopolymer concrete using deep-LSTM and various machine learning models offers significant benefits for the practical applications in the construction industry. The FA/GGBFS-based geopolymer concrete serves as an eco-friendly alternative to traditional concrete, as it incorporates industrial by-products to replace cement, resulting in a reduced carbon footprint and lower costs. However, a significant challenge is the high cost associated with the production of this type of concrete, largely due to the absence of specific codes and regulations to optimize its performance. A predictive model can help decrease these costs by optimizing various aspects of the manufacturing process, including mix design, quality control, process efficiency, and materials selection. Applying deep/machine learning models to predict the strength properties of geopolymer concrete offers a powerful tool for engineers and construction professionals. These models enable quick and accurate assessments of material performance during the design phase, leading to better-informed decisions. Additionally, predictive modeling minimizes the need for extensive laboratory testing, speeding up research and reducing costs for both researchers and construction projects utilizing geopolymer concrete.

## Limitations and recommendations of this study

This study aimed to compare the performance of deep LSTM with ML-based algorithms, including ANN, LSBoost, Bagging, and kNN, in predicting the f_c_ of FA/GGBFS-based GPC. Overall, the results were promising, with the exception of the kNN model. However, the applicability of these models is limited. The accuracy of the developed models relies heavily on the comprehensiveness and quality of the dataset used for training. While the dataset size is adequate compared to existing studies, a notable limitation is that it could be more extensive to improve the generalizability of the findings. For future research, it is recommended to expand the dataset size and input variables by including additional chemical compositions of the binders, various binder types, and different curing ages to better capture the variability. Furthermore, although the developed models showed strong capabilities in predicting the strength of FA/GGBFS-based GPC, expanding this study by including different concrete types could enhance both the reliability and applicability of the models. Future works could also involve comparing the performance of deep LSTM and ML-based algorithms across various concrete types, including engineered cementitious composites, traditional concretes, and self-compacting concretes.

## Conclusion

The reduction in CO_2_ emission and use of industrial wastes provide GPC with an eco-efficient type of concrete. The production of GPC is complicated due to various influencing factors, so taking all these influencing factors into account simultaneously using experimental approaches is challenging. A reliable and accurate prediction of f_c_ can provide time-saving, economical advantage, and performance optimization in mix preparation. Within this scope, there have been many studies related to the developed algorithms to estimate the f_c_ of FA/GGBFS-based GPC, especially about ANN. However, there has been no study about the f_c_ prediction of FA/GGBFS-based GPC by LSTM. Therefore, the devised LSTM model was compared with the most widely used algorithm, ANN, and rarely used algorithms, Bagging, LSBoost, and kNN, in the field of f_c_ prediction of FA/GGBFS-based GPC, for the first time. In this sense, the existing dataset having a similar mix design in the published literature was used. FA, GGBFS, fine and coarse aggregates, NaOH molarity, alkaline activators, SP, and curing temperature were used as input parameters, and based on Sensitivity Analysis, the chemical composition of GGBFS was found as the most effective parameter for the estimation of f_c_. The devised LSTM model outperformed ANN, Bagging, LSBoost, and kNN models in terms of performance metrics (R^2^ = 0.981) and error percentage distribution of all datasets, indicating the excellent potential of the LSTM model for the estimation of f_c_ of FA/GGBFS-based GPC. However, the fact that the ANN model had the highest accuracy with the R-squared value of 0.9912 in the training dataset also proves its accuracy in this field and explains its widespread use in previous studies. Moreover, the close accuracy percentage of LSBoost to both ANN and LSTM also showed the reliability of this type of ensemble model for this field. Besides, the evaluated correlation coefficients of Bagging for the training and testing dataset were higher than 0.8, indicating a reliable fit between actual and estimated results. On the other hand, the devised kNN model failed in the f_c_ estimation of FA/GGBFS-based GPC due to having so low accuracy for both training and testing.

The LSTM model was proposed in the current study as an alternative to predict the f_c_ of FA/GGBFS-based GPC. However, due to the reason that the high accuracy of ANN and LSBoost in the f_c_ estimation of FA/GGBFS-based GPC was proven in the current and previous studies, the combined use of the LSTM model with ANN or LSBoost can be examined in future studies. LSTM shows exceptional performance in both classification and regression tasks. However, their complex architecture, which consists of numerous layers and hidden units, requires significant computational resources, which can be a drawback. In contrast, LSTM excels at learning long-term dependencies, giving them an edge over traditional ANNs. On the other hand, ANNs have the advantage of being more resource-efficient, particularly in the classification phase, which can enhance overall performance. Thus, the ANN model may not be sufficient for long-term predictions and LSTM model may be impractical due to the necessity of large number of resources. Therefore, it is expected that combining LSTM and ANN model will strike the right balance between accuracy and efficiency. Besides, due to the high speed and simple structure of LSBoost algorithm, the combination of LSTM with LSBoost may also cause high performance.

## Supplementary Information

Below is the link to the electronic supplementary material.

Supplementary Material 1

## Data Availability

All data generated or analyzed during this study are available upon requested through corresponding author (Harun Tanyildizi).
